# Schema: metric learning enables interpretable synthesis of heterogeneous single-cell modalities

**DOI:** 10.1186/s13059-021-02313-2

**Published:** 2021-05-03

**Authors:** Rohit Singh, Brian L. Hie, Ashwin Narayan, Bonnie Berger

**Affiliations:** 1 Computer Science and Artificial Intelligence Laboratory, Massachusetts Institute of Technology, Cambridge, MA 02139 USA; 2 Department of Mathematics, Massachusetts Institute of Technology, Cambridge, MA 02139 USA

## Abstract

A complete understanding of biological processes requires synthesizing information across heterogeneous modalities, such as age, disease status, or gene expression. Technological advances in single-cell profiling have enabled researchers to assay multiple modalities simultaneously. We present Schema, which uses a principled *metric learning* strategy that identifies informative features in a modality to synthesize disparate modalities into a single coherent interpretation. We use Schema to infer cell types by integrating gene expression and chromatin accessibility data; demonstrate informative data visualizations that synthesize multiple modalities; perform differential gene expression analysis in the context of spatial variability; and estimate evolutionary pressure on peptide sequences.

## Introduction

High-throughput assays can now measure diverse cellular properties, including transcriptomic [1–3], genomic [4, 5], epigenomic [6–8], proteomic [9], functional [5], and spatial [10] data modalities. Excitingly, single-cell experiments increasingly profile multiple modalities simultaneously within the same experiment [5, 6, 9, 10], enabling researchers to investigate covariation across modalities; for instance, researchers can study epigenetic gene regulation by correlating gene expression and chromatin accessibility across the same population of cells. Importantly, since the underlying experiments provide us with multimodal readouts per cell, we do not need to integrate modalities across different populations of cells [11–17].

Simultaneous multimodal experiments present a new analytic challenge of synthesizing agreement and disagreement across modalities. For example, how should one interpret the data if two cells look similar transcriptionally but are different epigenetically? Moreover, given the rapid biotechnological progress that continues to enable novel measurement modalities and easier simultaneous multimodal profiling, a multimodal analysis paradigm should scale to massive single-cell datasets, be robust to noise and sparsity in the data, and be able to synthesize two or more arbitrary modalities in an interpretable way. Many existing methods, however, struggle with scalability, overfitting, or are specialized to specific multimodal tasks (such as just spatial transcriptomics [18–20] or only gene set estimation [21, 22]).

We therefore present Schema, a method that synthesizes multimodal data based on a conceptual framework that accommodates any number of arbitrary modalities. Schema draws from metric learning [23–26], the subfield of machine learning concerned with computing an accurate measure of similarity (equivalently, distance) on a dataset. Our critical insight is to interpret each modality as describing a measure of distance between the underlying cells; we can then newly formulate the synthesis problem as reconciling the information implied by these different distance measures.

Schema achieves this multimodal synthesis through an interpretable and principled quadratic programming formulation to compute the optimal reweighting of a modality’s features that maximizes its agreement with other modalities. Thus, a key advantage of our approach is that it provides feature weights that enable a researcher to understand where different modalities agree and where they do not. Our constrained optimization approach also improves Schema’s robustness to outliers and to overfitting.

In this study, we demonstrate the generality and utility of Schema. We synthesize RNA-seq and ATAC-seq modalities from multimodal data on 11,296 mouse kidney cells to infer cell types, with Schema enabling an 11% increase in accuracy over previously described approaches. On a dataset of 62,468 spatially resolved transcriptomes in the mouse cerebellum, we use Schema’s feature selection capabilities to identify genes differentially expressed between sparsely and densely packed granule cell neurons. We demonstrate how UMAP and t-SNE visualizations can be made more informative by infusing additional information, like cellular age, into the visualizations. Going beyond gene expression, we perform a feature selection analysis on a dataset of 62,858 T cells to estimate the locations and residues in the T cell receptor’s complementarity-determining region 3 (CDR3) important to its binding specificity. Schema is thus designed to support the continually expanding breadth of single-cell technologies while retaining the power, tunability, and interpretability required for effective exploratory analysis.

## Results

### Multimodal synthesis as metric learning

Before the advent of multimodal single-cell experiments, computational analysis has focused on variation within a single modality. In contrast, analysis of simultaneous multimodal single-cell experiments (where two or more modalities are available per cell) critically requires reasoning about information *across* modalities in a mutually consistent way. Our key intuition is that each modality gives us information about the biological similarity among cells in the dataset, which we can mathematically interpret as a modality-specific distance metric. For example, in RNA-seq data, cells are considered biologically similar if their gene expression profiles are shared; this may be proxied as the Euclidean distance between normalized expression vectors, with shorter distances corresponding to greater similarity.

**Fig. 1:**
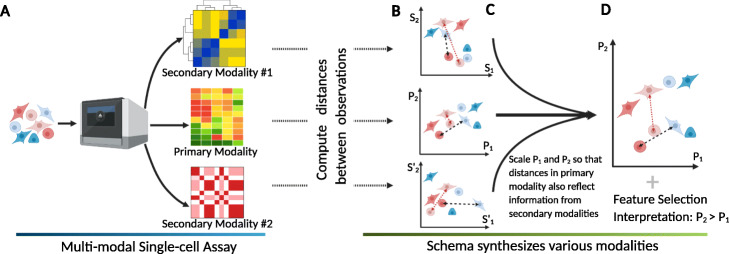
Integration of simultaneously assayed modalities using Schema. **a** Schema is designed for assays where multiple modalities are simultaneously measured for each cell. The researcher designates one high-confidence modality as the primary (i.e., reference) and one or more of the remaining modalities as secondary. **b** Each modality’s observations are mapped to points in a multi-dimensional space, with an associated distance metric that encapsulates modality-specific similarity between observations. Across the three graphs, the dashed and dotted lines indicate distances between the same pairs of observations. **c** Schema transforms the primary modality space by scaling each of its dimensions so that the distances in the transformed space have a higher (or lower, as desired) correlation with corresponding distances in the secondary modalities; arbitrary distance metrics are allowed for the latter. Importantly, the transformation is provably guaranteed to limit the distortion of the original space, thus ensuring that information in the primary modality is preserved. **d** The new point locations represent information synthesized from multiple modalities into a coherent structure. To compute the transformation, Schema weights features in the primary modality by their importance to its objective; we have found this feature selection aspect very useful in biological interpretation of its results

To synthesize these distance metrics, we draw inspiration from *metric learning* (Additional file 1: Text S3). Given a reference modality, Schema transforms this modality such that its Euclidean distances agree with a set of supplementary distance metrics from the other modalities, while also limiting the distortion of the original reference modality. Analyses on the transformed data will thus incorporate information from *all* modalities (Fig. 1). For instance, with RNA-seq data as the reference modality, Schema can transform the data so that it incorporates information from other modalities but limits the distortion from the original data so that the output remains amenable to standard RNA-seq analyses (e.g., cell-type inference, trajectory analysis, and visualization).

In our approach, the researcher starts by designating one of the modalities as the *primary* (i.e., reference) modality, consisting of observations that are mapped to points in a multi-dimensional space. In the analyses presented here, we typically designate the most informative or high-confidence modality as the primary or the reference modality, with RNA-seq being a frequent choice (Discussion). The coordinates of points in the primary modality are then transformed using information from *secondary* modalities. Importantly, the transformation’s complexity is constrained by limiting the distortion of the primary modality below a researcher-specified threshold. This acts as a regularization, preventing Schema from overfitting to other modalities and ensuring that the high-confidence information contained in the primary modality is preserved. We found this constraint to be crucial to successful multimodal syntheses. Without it, an unconstrained alignment of modalities using, for instance, canonical correlation analysis (CCA), a common approach in statistics for inferring information from cross-covariance matrices, or autoencoders, a deep learning approach for mapping multiple datasets to a shared latent space [27–30], is prone to overfitting to sample-specific noise, as we show in our results.

To see how Schema’s transformation synthesizes modalities, consider the case where the primary dataset is gene expression data. While the points close in Euclidean space are likely to be biologically similar cells with shared expression profiles, longer Euclidean distances are less informative. Schema’s constrained optimization framework is designed to preserve the information contained in short-range distances, while allowing secondary modalities to enhance the informativity of longer distances by incorporating, for example, cell-type metadata, differences in spatial density, or developmental relationships. To facilitate the representation of complex relationships between modalities, arbitrary distance metrics and kernels are supported for secondary modalities.

Schema’s measure of inter-modality alignment is based on the Pearson correlation of distances, which is optimized via a quadratic programming algorithm, for which further details are provided in “Methods.” An important advantage of Schema’s algorithm is that it returns coefficients that weight features in the primary dataset based on their agreement with the secondary modalities (for example, weighting genes in a primary RNA-seq dataset that best agree with secondary developmental age information). These feature weights enable greater interpretability into data transformations that is not immediately achievable by more complex, nonlinear transformation approaches [27–33]. We demonstrate this interpretability throughout our applications of Schema.

### Inferring cell types by synthesizing gene expression and chromatin accessibility

**Fig. 2:**
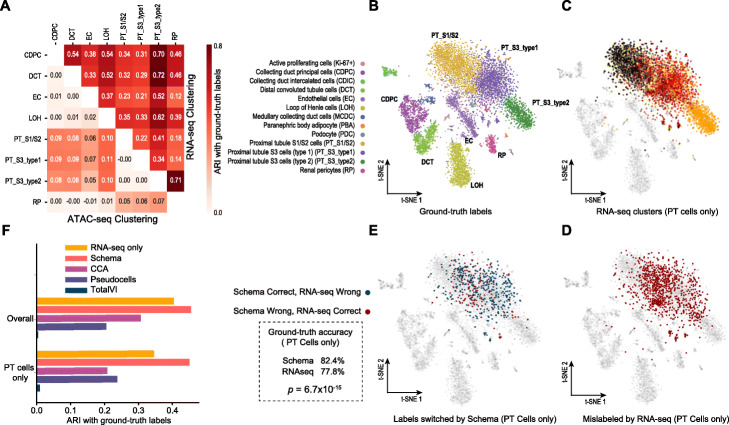
Synthesis of RNA-seq and ATAC-seq information leads to more accurate cell type inference. **a** Leiden clustering [34] of per-cell profiles results in greater agreement (measured as the adjusted Rand index, ARI) with ground truth cell type labels when featurizing cells by RNA-seq profiles alone compared to featurizing with ATAC-seq profiles alone. ATAC-seq does provide relatively more information when distinguishing PT cells. **b** Ground truth labels from Cao et al. [6]. **c**–**e** To assess the ground truth accuracy of Leiden clustering, we assigned each cluster to the cell type most frequently seen in the ground truth labels of its members. Clusters where labels are more mixed will thus have lower accuracy. Clustering on RNA-seq profiles alone (**c**,**d**) results in many PT cells assigned to such clusters. Schema synthesis of RNA- and ATAC-seq features, followed by Leiden clustering (**e**), results in significantly greater concordance with ground truth on PT cell types when compared to Leiden clustering on the RNA-seq features alone (one-sided binomial test, *p* = 6.7 × 10^− 15^). **f** ARIs of clusters from Schema-synthesized data are higher, especially for PT cells. Synthesizing the modalities using canonical correlation analysis (CCA), totalVI (an autoenconder-based deep learning approach), or a “pseudocell” approach described in the original study (see Methods) results in lower ARI scores

We first sought to demonstrate the value of Schema by applying it to the increasingly common and broadly interesting setting in which researchers simultaneously profile the transcriptome and chromatin accessibility of single cells [6]. Focusing on cell type inference, a key analytic step in many single-cell studies, we applied Schema on a dataset of 11,296 mouse kidney cells with simultaneously assayed RNA-seq and ATAC-seq modalities and found that synthesizing the two modalities produces more accurate results than using either modality in isolation (Fig. 2f; Additional file 1: Figure S3).

With RNA-seq as the primary (i.e., reference) dataset and ATAC-seq as the secondary, we applied Schema to compute a transformed dataset in which pairwise RNA-seq distances among cells are better aligned with distances in the ATAC-seq peak counts data while retaining a very high correlation with primary RNA-seq distances (≥ 99%, “Methods”). We then clustered the cells by performing Leiden community detection [34] on the transformed dataset and compared these clustering assignments to the Leiden clusters obtained without Schema transformation. We measured the agreement of these fully automated clusterings with expertly defined ground truth cluster labels (from Cao et al. [6]), quantifying this agreement with the adjusted Rand index (ARI), which has a higher value if there is greater agreement between two sets of labels. Leiden clustering on Schema-transformed data better agrees with the ground truth annotations of cell types (ARI of 0.46) than the corresponding Leiden cluster labels using just RNA-seq or ATAC-seq datasets individually (ARIs of 0.40 and 0.04, respectively, Fig. 2f). Here, Schema facilitated a biologically informative synthesis despite limitations of data quality or sparsity in the ATAC-seq secondary modality. We observed that using only ATAC-seq data to identify cell types leads to poor concordance with ground truth labels (Additional file 1: Figure S3A), likely because of the sparsity of this modality (for example, only 0.28% of the peaks were reported to have non-zero counts, on average); this sparsity was also noted by the original study authors.

To further analyze why combining modalities improves cell type clustering, we obtained Leiden cluster labels using either the RNA-seq or the ATAC-seq modalities individually. We then evaluated these cluster assignments by iterating over subsets of the data, each set covering only a pair of ground truth cell types and used the ARI score to quantify how well the cluster labels distinguished between the two cell types. While RNA-seq clusters have higher ARI scores overall, indicating a greater ability to differentiate cell types, ATAC-seq does display a relative strength in distinguishing proximal tubular (PT) cells from other cell types (Fig. 2a). PT cells are crucial to kidney function, with the specific PT cell sub-types playing distinct roles in, for instance, glucose reabsorption [35]. They are also the most numerous cells in this dataset and many of the misclassifications in the RNA-seq based clustering relate to these cells (Fig. 2b–d). When the two modalities are synthesized with Schema, a significant number of these PT cells are correctly assigned to their ground truth cell types (one-sided binomial test, *p* = 6.7 × 10^− 15^), leading to an overall improvement in clustering quality (Fig. 2e). Furthermore, upon analyzing Schema’s feature selection output, we found that the genes it up-weighted in the primary RNA-seq modality were differentially expressed in PT cells (one-sided *t*-test, FDR *q* < 0.01 for each of the three PT cell types), thus emphasizing the RNA-seq subspace where support from the secondary modality signal was strongest. These genes (the top hits are *Pnisr*, *Ankrd11*, and *Kmt2c*) are enriched for regulation of macromolecule metabolic process (GO:0060255, FDR *q* = 0.0103) and regulation of nitrogen compound metabolic process (GO:0051171, FDR *q* = 0.0133).

### Schema’s constrained data synthesis outperforms unconstrained approaches

In general, synthesis of multimodal data can also be done by statistical techniques like canonical correlation analysis (CCA) or deep learning architectures that represent multiple modalities in a shared latent space [27–33]. A key conceptual advance of Schema over these approaches is its emphasis on limiting the distortion of the high-confidence reference modality, allowing it to extract signal from the lower-confidence secondary modalities without overfitting to their noise and artifacts. Intuitively, the synthesis of two modalities requires the identification of a subspace (or latent space) in each modality that aligns well with the other. Due to noise and artifacts, an unconstrained approach may overfit by identifying a pair of subspaces that align well but are biologically uninformative. In contrast, Schema’s constrained optimization formulation, combined with the use of a high-confidence modality as the primary, ensures that any possible alignment will use only a biologically informative subspace of the primary modality and thus guides the quadratic programming optimizer towards correspondingly informative subspaces in the other modalities. To demonstrate the importance of this constrained approach, we evaluated the performance of CCA and totalVI [30] in integrating the RNA-seq and ATAC-seq modalities (Fig. 2f). We applied CCA to synthesize the two modalities and performed Leiden clustering on the resulting dataset, finding its overlap with the ground truth labels (ARI of 0.31) to be lower than that from Schema’s synthesis (0.46). Indeed, this is a lower ARI than is achievable just with RNA-seq data (0.40), indicating that the CCA-based synthesis may be overfitting to the sparse and noisy ATAC-seq data.

To evaluate an autoencoder-based synthesis of these modalities, we applied scVI [27] and totalVI to compute per-modality and dual-modality latent space representations, respectively (Methods). We performed Leiden clustering in the autoencoder latent spaces and evaluated the clustering’s overlap with ground truth labels. We first verified that the single-modality latent space representations did lead to Leiden clusters of comparable quality as had previously been observed from Leiden clustering on the raw data (ARIs of 0.365 and 0.038 for scVI-generated representations of RNA-seq and ATAC-seq data, respectively). However, the dual-modality shared-space representation from totalVI produced a Leiden clustering (Additional file 1: Figure S3B) that had a low overlap with the ground truth (ARI of 0.0043). We hypothesize that the sparsity and low signal-to-noise ratio here in the ATAC-seq modality led totalVI to a latent space representation that corresponds to low biological-information subspaces of the two modalities, rather than their respective high information subspaces. We note that we were able to achieve better performance with totalVI when applying the same procedure to a synthetic, less-noisy secondary modality consisting of partially randomized RNA-seq observations (Methods).

While these CCA and autoencoder results were likely due to overfitting, the Schema-based synthesis constrains the ATAC-seq modality’s influence, enabling us to extract additional signal provided by ATAC-seq while preserving the rich information provided by the transcriptomic modality. We believe that this regularization offered by Schema’s constrained optimization formulation is a key advantage that will be crucial in multimodal single-cell data synthesis. We also note that Schema offers additional advantages: unlike CCA, it can incorporate more than two modalities simultaneously and, unlike totalVI, its synthesis is interpretable, revealing a more accurate characterization of PT cells.

### Schema highlights secondary patterns while preserving primary structure

Another powerful use of Schema is to infuse information from other modalities into RNA-seq data while limiting the data’s distortion so that it remains amenable to a range of standard RNA-seq analyses. Since widely used visualization methods such as UMAP [36] do not allow a researcher to specify aspects of the underlying data that they wish to highlight in the visualization, we sought to apply Schema to improve the informativity of single-cell visualizations. We leveraged Schema to highlight the age-related structure in an RNA-seq dataset of *Drosophila melanogaster* neurons [3] profiled across a full lifespan, while still preserving most of the original transcriptomic structure. We chose RNA-seq as the primary modality and temporal metadata (cell age) as the secondary modality, configuring Schema to maximize the correlation between distances in the two while constraining the distortions induced by the transformation (Methods). We then visualized the transformed result in two dimensions with UMAP.

**Fig. 3:**
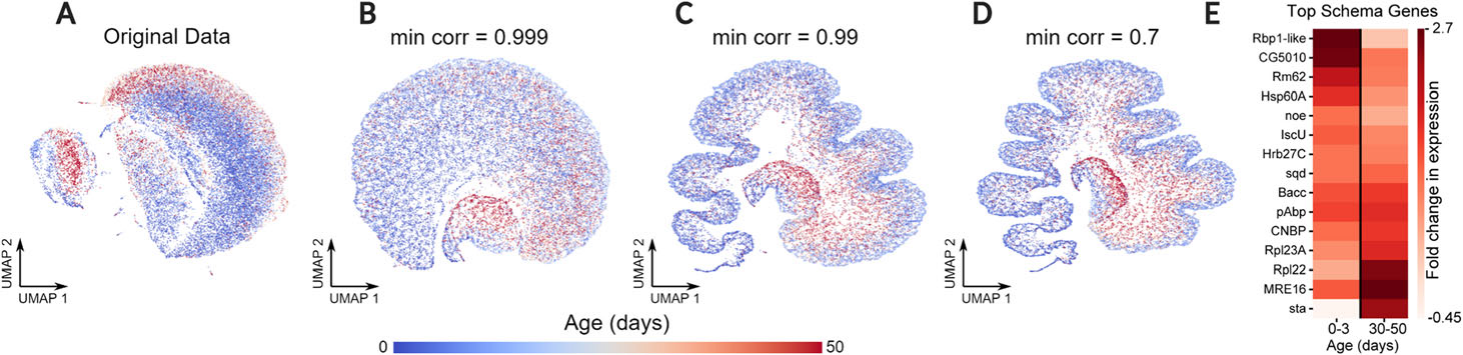
Incorporating temporal metadata into UMAP visualizations of aging neurons captures developmental changes. UMAP visualization of RNA-seq profiles of *D. melanogaster* neurons at 0, 1, 3, 6, 9, 15, 30, and 50 days after birth, representing the full range of a typical *D. melanogaster* lifespan. The transcriptomic data (primary modality) was transformed to a limited extent using Schema by correlating it with the temporal metadata (secondary modality) associated with each cell. **a** UMAP visualization of the original transcriptomic data. **b–d** Visualizations of transformed data with increasing levels of distortion. As the value of the minimum correlation constraint *s* approaches 1, the distortion of the original data is progressively limited. Decreasing *s* results in a UMAP structure that increasingly reflects an age-related trajectory. **e** Feature selection interpretation of Schema’s transformation. In synthesizing the two modalities, Schema up-weights genes (top 15 shown here) that are differentially active at the start or end of the time-course. For clarity, the set of genes has been reordered by the difference in their early and late-stage expression

While some age-related structure does exist in the original data, Schema-based transformation of the data more clearly displays a cellular trajectory consistent with biological age (Fig. 3). Importantly, revealing this age-related structure required only a limited distortion of the data, corresponding to relatively high values (≥ 0.99) of the minimum correlation constraint (Fig. 3c).

Analysis of Schema’s feature selection indicated an up-weighting of genes differentially expressed at the start or end of the aging process (Fig. 3e), with genes implicated in cell organization/biogenesis [37] (e.g., *Rm62, CG5010* and *IscU*) active at the start while ribosomal genes like *Rpl22* and *Rpl23A* were active at the end. We also confirmed that there was a significant overlap between Schema’s highest-ranked genes and those found by a standard differential expression test between timepoints (one-sided binomial test, FDR *q* < 10^− 21^ for the 1-, 30-, and 50-min subsets). To additionally verify that Schema was infusing additional age-related structure into RNA-seq data, we performed a diffusion pseudotime analysis of the original and transformed datasets and found that the Spearman rank correlation between this pseudotime estimate and the ground truth cell age increased from 0.365 in the original data to 0.405 and 0.436 in the transformations corresponding to minimum correlation constraints of 0.999 and 0.99, respectively.

We note that the constrained optimization of Schema was again important to retaining biological signal during the synthesis: in comparison, an unconstrained synthesis by CCA led to a lower pseudotime correlation (0.059) than seen in the original RNA-seq dataset; the corresponding CCA-based UMAP visualization was also less clear in conveying the cellular trajectory (Additional file 1: Figure S6). Schema thus enables visualizations that synthesize biological metadata, while preserving much of the distance-related correlation structure of the original primary dataset. With Schema, researchers can therefore investigate single-cell datasets that exhibit strong latent structure (e.g., due to metadata like age or spatial location), infusing this secondary information into the primary RNA-seq modality. We recommend specifying a high minimum correlation constraint (e.g., 0.99) during the synthesis (Discussion), having observed that only a small transformation of the RNA-seq data is needed to make the latent structure visible.

### Spatial density-informed differential expression among cerebellar granule cells

In addition to cell type inference, another important single-cell analysis task that stands to benefit from multimodal synthesis is the identification of differentially expressed marker genes. To perform differential expression analysis with Schema, RNA-seq data should be used as the primary modality, while the distance metrics of the secondary modalities specify how cells should be differentiated from each other. We applied Schema to spatial transcriptomics data, another increasingly important multimodal scenario, here encompassing gene expression, cell-type labels, and spatial location.

**Fig. 4:**
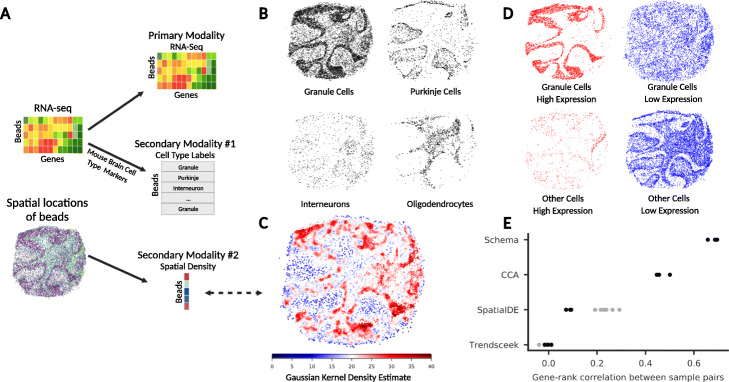
Schema identifies a gene set in granule neurons whose expression covaries with spatial cellular density. **a** Rodriques et al. [10] simultaneously assayed spatial and transcriptomic modalities in mouse cerebellum tissue (data from puck 180430_1 is shown here). In addition, they labeled beads (each corresponding to a transcriptome) with a putative cell type by comparing gene expression profiles with known cell-type markers. **b** Spatial distribution of the most common cell types in the tissue: granule cells, Purkinje cells, interneurons, and oligodendrocytes. Note the variation in spatial density for granule cells. **c** We quantified this spatial density variation by computing a two-dimensional Gaussian kernel density estimate, with cells in dense regions assigned a higher score. **d** Schema is able to identify a set of genes that are highly expressed only in densely packed granule cells. The four figures here show mutually disjoint sets of cells: granule cells with high expression of the gene set, granule cells with low expression of the gene set, other cells with high expression, and other cells with low expression. Here, a cell is said to have high expression of the gene set if the cell’s loading on this gene set ranks in the top quartile. **e** Schema’s results are robust across biological replicates. Across three replicates, we evaluated the consistency of gene rankings computed by Schema, canonical correlation analysis (CCA), SpatialDE, and Trendsceek. The black points indicate the Spearman rank correlation of gene scores across pairs of replicates. We needed to adapt SpatialDE and Trendsceek for this task by first applying them separately on granule and non-granule cells and then combining the results (Methods); here, the black and gray points indicate the cross-replicate correlations of the final and intermediate gene rankings, respectively

We obtained Slide-seq data containing 62,468 transcriptomes that are spatially located in the mouse cerebellum. In the original study, these transcriptomes were assigned to putative cell types (noting that these transcriptomes are not guaranteed to be single cell), and thus cell types are located throughout the tissue [10, 38]. Interestingly, we observed spatial density variation for certain cell types; specifically, transcriptomes corresponding to granule cell types are observed in regions of both high and low spatial density (Fig. 4b in this paper; also Fig. 2b of Rodriques et al. [10]).

Schema’s feature selection capabilities could thus identify genes that are differentially expressed in granule cells in high density areas versus granule cells in low density areas. Schema is well suited to the constrained optimization setting of this problem: we optimize for genes expressed specifically in granule cells *and* in dense regions, but not all granule cells are in dense regions and not all cells in dense regions are granule cells. We specified RNA-seq data as the primary modality and spatial location and cell-type labels as the secondary modalities. In the spatial location modality, the distance metric was defined such that two cells are similar if their spatial neighborhoods have similar density (Methods).

The densely packed granule cell genes identified by Schema are strongly enriched for GO terms and REACTOME pathways [39] related to signal transmission, e.g., ion-channel transport (REACTOME FDR *q* = 1.82 × 10^− 3^), ion transport (GO:0022853, FDR *q* = 1.8 × 10^− 17^), and electron transfer (GO:009055, FDR *q* = 2.87 × 10^− 11^). This finding suggests potentially greater neurotransmission activity within these cells (Additional file 1: Figures S9–S10, Text S6; Additional file 2: Table S2–S3).

### Schema outperforms alternative methods for spatial transcriptomic analysis

We sought to benchmark our method by comparing the robustness of Schema’s results with those based on canonical correlation analysis (CCA) and with two methods specifically intended for spatial transcriptomics, namely SpatialDE [18] and Trendsceek [19].

An important point is that CCA, SpatialDE, and Trendsceek are less general than Schema and therefore require non-trivial modifications to approximately match Schema’s capabilities. CCA is limited in that it can correlate only two datasets at a time, whereas here we seek to synthesize *three* modalities: gene expression, cell-type labels, and spatial density. We adapted CCA by correlating two modalities at a time and combining the sub-results (Methods). In the case of SpatialDE and Trendsceek, their unsupervised formulation does not allow the researcher to specify the spatial features to pick out (we focus on spatial *density* variation). To adapt these, we collated their results from separate runs on granule and non-granule cells (Methods). Notably, the ad hoc modifications required to extend existing methods beyond two modalities underscore the benefit of Schema’s general analytic formulation that can be naturally extended to incorporate any number of additional data modalities.

Reasoning that a robust computational approach should return consistent results across biological replicates, we evaluated the stability and quality of each spatial transcriptomic technique by comparing its results on three replicate samples of mouse cerebellum tissue (coronal sections prepared on the same day [10]; pucks 180430_1, 180430_5, 180430_6) (Methods). While both Schema and CCA identify a gene set that ostensibly corresponds to granule cells in dense regions (Fig. 4d; Additional file 1: Figure S4), the gene rankings computed by Schema are more consistently preserved between pairs of replicates than those computed by CCA, with the median Spearman rank correlation between sample pairs being 0.68 (Schema) versus 0.46 (CCA). Likewise, with Schema, 69.1% of enriched GO biological-process terms are observed in all three samples and 78% are in at least two samples. The corresponding numbers for CCA were 35.7% and 59.5%, respectively (FDR *q* < 0.001 in all cases). We thus find that Schema’s results are substantially more robust across the three replicates. Compared to CCA’s unconstrained synthesis, Schema’s constrained formulation avoids overfitting to sample-specific noise, enhancing its robustness (Methods; Fig. 4e; Additional file 1: Figure S5).

When performing the same gene list robustness analysis with SpatialDE and Trendsceek, while also looking at the stability of their gene rankings specific to the precursor cell type (gray points in Fig. 4e), we found that SpatialDE produced slightly more stable gene rankings than Trendsceek, with median sample-pair correlations of 0.089 and − 0.002, respectively, but these were still lower than those for Schema. We also observed that SpatialDE and Trendsceek had substantially longer running times and we performed our analysis of the two methods on subsets of the overall dataset (see “Schema can scale to massive single-cell datasets” for precise runtime and memory usage). These results demonstrate the robustness and efficiency of Schema’s supervised approach.

### Beyond gene expression: Schema reveals CDR3 segments crucial to T cell receptor binding specificity

To further demonstrate the generality of Schema, we applied it to synthesize data modalities beyond gene expression. We integrated single-cell multimodal proteomic and functional data with Schema to better understand how sequence diversity in the hypervariable CDR3 segments of T cell receptors (TCRs) relates to antigen binding specificities [40]. De novo design of TCRs for an antigen of interest remains a pressing biological and therapeutic goal [41, 42], making it valuable to identify the key sequence locations and amino acids that govern the binding characteristics of a CDR3 segment. Towards this end, we analyzed a single-cell dataset that recorded clonotype data for 62,858 T cells and their binding specificities against a panel of 44 ligands [5] and used Schema’s feature selection capabilities to estimate the sequence locations and residues in the CDR3 segments of α and β chains important to binding specificity.

To estimate location-specific selection pressure, we ran Schema with the CDR3 peptide sequence data as the primary modality and the binding specificity information as the secondary modality, performing separate runs for α and β chains. In the primary modality, each feature corresponds to a CDR3 sequence location and we used the Hamming distance metric between observations (i.e., the number of locations at which two sequences differ, see Methods). Schema assigned relatively low feature weights to the location segments 3–9 (in α chain CDR3) and 5–12 (in β chain CDR3), suggesting those regions can tolerate greater sequence variability while preserving binding specificity.

**Fig. 5:**
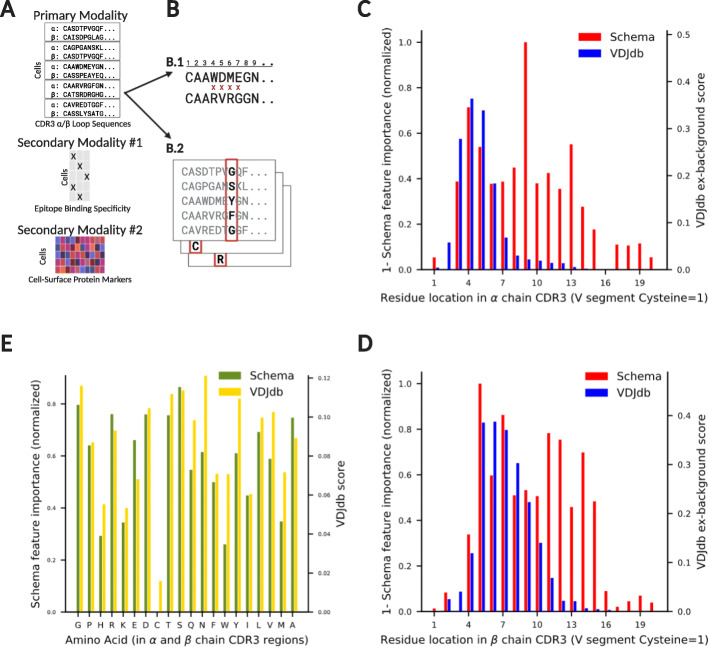
Schema reveals the locations and amino acids important in preserving binding specificity of T cell receptor CDR3 regions (https://help.biorender.com/en/articles/3619405-how-do-i-cite-biorender). **a** We analyzed a multimodal dataset from 10x Genomics [5] to understand how a T cell receptor’s binding specificity relates to the sequence variability in the CDR3 regions of its α and β chains. The primary modality consisted of CDR3 peptide sequence data which we correlated with the secondary modality, the binding specificity of the cell against a panel of 44 epitopes. We optionally synthesized an additional modality, proteomic measurements of 12 cell-surface marker proteins, as a use-case of incorporating additional information (Methods). **b** We performed two Schema analyses: (*B.1*) To infer location-wise selection pressure, each feature of the primary modality corresponded to a location in CDR3 sequence; (*B.2*) To infer amino acid selection pressure, the primary modality was the Boolean vector of residues observed at a specific sequence location; we aggregated over an ensemble of Schema runs across various locations. **c**, **d** Schema identifies sequence locations 3–9 (α chain) and 5–12 (β chain) as regions where sequences can vary with a comparatively modest impact on binding specificity. We compared Schema’s scores to statistics computed from motifs in VDJdb. Here, we have inverted the orientation of Schema’s weights to align them with the direction of VDJdb weights. **e** Schema and VDJdb agree on the relative importance of amino acids in preserving binding specificity (Spearman rank correlation = 0.74, two-sided *t*-test *p* = 2 × 10^− 4^). The low weight assigned to cysteine is likely due to its infrequent occurrence in the data

To evaluate these results, we compared them to estimates based on CDR3 sequence motifs sourced from VDJdb [43], a curated database of TCRs with known antigen specificities. In VDJdb, TCR motifs are scored using an adaptation of the relative entropy algorithm [44] by Murugan et al. that assigns a score for each location and amino acid in the motif. We aggregated these scores into a per-location score (Methods), allowing a comparison with Schema’s feature weights (Fig. 5). While the comparison at locations 11–20 is somewhat complicated by VDJdb having fewer long sequences (Methods), there is agreement between Schema and VDJdb estimates on locations 1–10 where both datasets have good coverage (Spearman rank correlations of 0.38 and 0.92 for the α and β chains, respectively; Fig. 5c, d). We note that weight estimation using Schema required only a single multimodal dataset; in contrast, extensive data collection, curation, and algorithmic efforts underlie the VDJdb annotations. The latter covers multiple experimental datasets, including the 10x Genomics dataset [5] we investigated here; we saw similar results when comparing against an older version of VDJdb without this dataset.

Next, we used Schema to investigate the selection pressure on amino acids present in the variability-prone locations identified above (Methods). We first selected a sequence location (e.g., location 4 in α chain CDR3) and constructed a primary modality where each cell was represented by a one-hot encoding of the amino acid at the location (i.e., a 20-dimensional Boolean vector). The secondary modality was binding specificity information, as before. We performed separate Schema runs for each such location of interest on the two chains, computing the final score for each amino acid as the average score across these runs. These scores are in good agreement with the corresponding amino acid scores aggregated from the VDJdb database (Spearman rank correlation = 0.74, two-sided *t*-test *p* = 2 × 10^− 4^). The residue and location preferences estimated here can directly be used in any algorithm for computational design of epitope-specific CDR3 sequences to bias its search towards more functionally plausible candidate sequences.

Schema’s ability to efficiently synthesize arbitrarily many modalities, with their relative importance at the researcher’s discretion, allows information that might otherwise be set aside (e.g., metadata like batch information, cell line, or donor information) to be effectively incorporated, enhancing the robustness and accuracy of an analysis. In Methods, we exemplify this use-case on the TCR dataset by incorporating measurements of cell-surface markers as an additional secondary modality, hypothesizing that cell-surface protein levels should be unrelated to V(D)J recombination variability.

### Additional demonstrations

Applying Schema on a mouse gastrulation dataset [45] consisting of 16,152 epiblast cells split over three developmental timepoints and with two replicates at each timepoint, we performed differential expression analysis while simultaneously accounting for batch effects and developmental age, and evaluated its results alongside those from MOFA+, a recently introduced single-cell multimodal analysis technique [22, 46] (Additional file 1: Figure S1, Text S1). We also used Schema to study cell differentiation by synthesizing spliced and unspliced mRNA counts in a dataset of 2930 mouse dentate gyrus cells [47]. As in standard RNA velocity analyses, correlating spliced and unspliced counts in a cell picks up on the time derivative of a cell’s expression state and thus illuminates the cell differentiation process. Schema’s results agree with those from the dedicated RNA velocity tool scVelo [48], and we also demonstrate how Schema can be used to infuse velocity information into a t-SNE visualization (Additional file 1: Figure S2, Text S2).

### Schema can scale to massive single-cell datasets

We have designed Schema to process large single-cell datasets efficiently, with modest memory requirements. On average, Schema processes data from a Slide-seq replicate (three modalities, 20,823 transcriptomes × 17,607 genes) in 34 min, requiring less than 5 GB of RAM in the process (Additional file 1: Table S1). The runtime includes the entire set of Schema sub-runs performed over an ensemble of parameters, as well as the time taken for the preprocessing transformation.

Schema’s efficiency stems from our novel mathematical formulation. Deviating from standard metric learning approaches, we formulate the synthesis problem as a quadratic program optimization, which can be solved much faster than the semidefinite program formulations typically seen in these approaches (Additional file 1: Text S3). Additionally, while the full Schema algorithm has quadratic scalability in the number of cells, our formulation allows us to obtain good approximations with *provably* bounded error using only a logarithmic subsample of the dataset (Additional file 1: Text S5), enabling *sublinear* scalability in the number of cells that will be crucial as multimodal datasets increase in size. These subsampling techniques can also leverage diversity-preserving data sketching techniques [49, 50] that may empirically lead to greater representation of rare cell types in the Schema analysis.

## Discussion

We designed Schema to be a powerful approach to multimodal data analysis. Schema is based on an elegant conceptual formulation in which each modality is defined using a distance metric. A key conceptual advance of this work is to formulate the synthesis task as a constrained optimization problem, allowing Schema to robustly accommodate noisy and sparse modalities. The strength of this intuition enables analysis of an arbitrary number of modalities and applicability to any modality, so long as it is possible to define an appropriate distance metric. Importantly, the synthesis is interpretable, with Schema identifying the features of the primary (i.e., reference) modality that drive the integration.

Our approach enables the researcher to supervise the synthesis by choosing which modality to transform, the degree to which it can be distorted, and the desired level of agreement between modalities. While existing methods like Seurat v3 [16] and LIGER [17] are designed for unsupervised discovery of common patterns across experiments, Schema’s supervised formulation facilitates a broader set of investigations, enabling us to not only infer cell types and identify gene sets but also, for instance, rank amino acids by selection pressure.

When choosing a primary modality, we generally recommend selecting the most high-confidence modality or the one for which feature selection will be most informative, though it can sometimes be productive to integrate insights across multiple invocations of Schema with varying primary modality choices. In many of our demonstrations, we chose RNA-seq as the primary modality since it is often the modality where preprocessing and normalization are best understood, boosting our confidence in it; additionally, transformed RNA-seq data lends itself to a variety of downstream analyses. Once a primary modality has been designated, Schema can synthesize an arbitrary number of secondary modalities with it. In contrast, methods designed around pairwise modality comparison need ad hoc adaptations to accommodate additional modalities. Schema’s approach is advantageous not only for datasets with more than two modalities [5, 51] but also in cases where metadata (e.g., batch information and cell age) can be productively incorporated as additional modalities.

Intuitively, our correlation-based alignment approach has parallels to kernel canonical correlation analysis (kernel CCA), a generalization of CCA where arbitrary distance metrics can be specified when correlating two datasets. While Schema offers similar flexibility for secondary modalities, it limits the primary modality to Euclidean distances. Introducing this restriction enhances scalability, interpretability, and robustness. Unlike kernel CCA, the optimization in Schema operates on matrices whose size is independent of the dataset’s size, enabling it to scale sub-linearly to massive single-cell datasets. Also, the optimal solution is a scaling transform that can be naturally interpreted as a feature-weight vector. Perhaps most importantly, Schema differs from kernel CCA in performing a constrained optimization, thus reducing the distortion of the primary dataset and ensuring that sparse and low-confidence secondary datasets do not drown out the primary signal.

The constrained optimization in Schema acts as regularization, helping ensure that the computed transformation and feature selection remain biologically meaningful. By choosing a high-confidence modality as the primary modality and bounding its distortion when incorporating the secondary modalities, Schema enables information synthesis while retaining high-confidence insights. This bound on the distortion is an important parameter, directly controlling how much the secondary modalities inform the primary dataset; values approaching 1 will increasingly limit the influence of the secondary modalities. Therefore, we recommend that studies using Schema for feature selection should aggregate the results over a range of values of this parameter while analyses that utilize only a single parameter should keep it high (≥ 0.9, the default setting in our implementation is 0.99) to preserve fidelity with the original dataset (Methods). If sufficient data is available, cross-validation can also be used to tune this parameter. We strongly recommend that studies with a single parameter should report the value of this parameter alongside their results.

Interesting future methodological work could explore alternative formulations of the Schema objective, potentially including more complex nonlinearities than our quadratic program formulation. Schema can also be used in conjunction with data-integration methods [16, 17] designed for cases where each modality was assayed on different cells: after a cross-modality cell-to-cell correspondence has been computed, Schema can be applied to interpret the integrated data. It can also guide further biological experiments that profile only the highly weighted features based on other data modalities, enabling efficient, targeted follow-up analysis.

Given the current pace of biotechnological development, we anticipate that high-throughput experiments, and their conclusions, will increasingly rely on more than one data modality, underscoring the importance of Schema and its conceptual framework. Schema is publicly available for use at http://schema.csail.mit.edu and as the Python package *schema_learn*.

## Methods

### Correlation-based alignment and quadratic programming optimization

Underlying our definition of the alignment of metrics is the intuitive notion that metrics are similar if the ordering of pairwise distances between the two metrics are close. A proxy for measuring this alignment is the Pearson correlation coefficient. For Schema, the goal is thus that pairwise distances in the transformed space be highly correlated with pairwise distances under each metric.

One of the advantages of the Pearson correlation coefficient is that it is amenable to optimization via *quadratic programming* (QP)*.* QP is a generalization of linear programming, allowing a quadratic objective function. We learn a *scaling transformation u* (Additional file 1: Text S3) on the primary dataset *X* such that the pairwise distances of the transformation *u* ∗ *x*_*i*_ (where ∗ denotes coordinate-wise multiplication, for each *x*_*i*_ ∈ *X*) are highly correlated with the pairwise distances in the secondary modalities. We codify our intuition of the importance of the primary dataset by requiring that the correlation of transformed pairwise distances with the original dataset be higher than some researcher-specified threshold. The scaling transformation has the appealing property of being interpretable as a *feature selection*: the higher the coordinate *u*_*i*_, the more important that coordinate is for alignment. Thus, by selecting the top coordinates by their weights, we can access the genes most important for aligning the modalities.

### Mathematical formulation

Suppose we have *N* observations across *r* datasets *D*_*j*_, *j* = 1, 2, …, *r*, where ${D}_j=\left\{{x}_i^{(j)}:i=1,2,\dots, N\right\}$ contains data (categorical or continuous) for each observation. We will refer to *D*_1_ as the *primary* dataset and the rest as secondary. Each dataset’s dimensionality and domain may vary. In particular, we assume *D*_1_ is *k*-dimensional. Each dataset *D*_*j*_ should also have some notion of distance between observations attached to it, which we will denote *ρ*_*j*_, so ${\rho}_j\left({x}_n^{(j)},{x}_m^{(j)}\right)$ is the distance between observations *n* and *m* in *D*_*j*_. Since our entire framework below deals in *squared* distances, for notational convenience we will let *ρ*_*j*_ be the squared distances between points in *D*_*j*_; also, we drop the superscript in ${x}_j^{(1)}$ when referring to the primary dataset *D*_1_ and its data.

The goal is to find a transformation *Ω* with *Ω*(*D*) generating a dataset *D*^∗^ such that the Euclidean metric *ρ*^∗^ on *D*^∗^ aligns the various metrics *ρ*_*j*_, each informed by its respective modality. Non-Euclidean primary distance metrics *ρ*_1_ are allowed if they can be computed as a sum of *k* terms, one for each feature (e.g., Hamming distance). We emphasize that none of the secondary *ρ*_*j*_ need to be Euclidean. This setup is quite general, and we now specify the form of the transformation *Ω* and the criteria for balancing information from the various metrics. Here, we limit *Ω* to a *scaling transform*. That is, *Ω*(*D*) = {*diag*(*u*)*x* : *x* ∈ *D*} for some *u* ∈ *R*^*k*^ and *diag*(*u*) is a *k* × *k* diagonal matrix with *u* as its diagonal entries. Then, the squared distance between points under the transformation is given by

$$ {\rho}^{\ast}\left({x}_n,{x}_m\right)={\left\Vert \mathit{\operatorname{diag}}(u){x}_n-\mathit{\operatorname{diag}}(u){x}_m\right\Vert}^2=\mathit{\operatorname{diag}}(w){\left\Vert {x}_n-{x}_m\right\Vert}^2 $$

where *w* is the element-wise square of *u*, i.e., ${w}_i={u}_i^2$. The scaling transform *u* acts as a feature-weighting mechanism: it chooses the features of *D*_1_ that align the datasets best (i.e., *u*_*i*_ being large means that the *i*th coordinate of *D*_1_ is important). We note here that a natural extension would be allowing *general linear* transformations for *Ω*; however, in that context, the fast framework of quadratic programming would need to be substituted for the much slower framework of semidefinite programming.

Here, our approach to integration between the metrics *ρ*_*j*_ is to learn a metric *ρ*^∗^ that aligns well with all of them. Our measure of the alignment between *ρ*^∗^ and *ρ*_*j*_ is given by the Pearson correlation between pairwise squared distances under two metrics. Intuitively, maximizing the correlation coefficient encourages distances under *ρ*^∗^ to be large when the corresponding *ρ*_*j*_ distances are large and vice versa. This can be seen from the expression

1 $$ Corr\left({\rho}^{\ast },{\rho}_j\right)=\frac{Cov\left({\rho}^{\ast },{\rho}_j\right)}{{\left( Var\left({\rho}^{\ast}\right)\  Var\left({\rho}_j\right)\right)}^{1/2}} $$

To deal with multiple modalities, we try to maximize the correlation between *ρ*^∗^ and the distances on each of the metrics, allowing the user to specify how much each modality should be weighted. We also allow a hard constraint, whereby the correlation between the pairwise distances in the transformed data and in the primary dataset is lower-bounded. Our goal is thus to find

2 $$ {\displaystyle \begin{array}{c}\left\{{\sum}_{j=2}^r{\gamma}_j Corr\left({\rho}^{\ast }(w),{\rho}_j\right)\ \right\}\ \\ {} subject\ to\\ {} Corr\left({\rho}^{\ast }(w),{\rho}_1\right)\ge s\end{array}} $$

where *γ*_*j*_ and *s* are hyperparameters that determine the importance of the various metrics. We have also highlighted that *ρ*^∗^ is a function of *w* and is determined entirely by the solution to (2). In the rest of our discussion, we will abuse notation and primarily use *w*, rather than *ρ*^∗^, to refer to the optimal metric. The machinery of *quadratic programming* makes this optimization feasible.

### Setting up the quadratic program

As motivated above, quadratic programming (QP) is a framework for constrained convex optimization problems that allows a quadratic term in the objective function and linear constraints. The general form is

3 $$ {\displaystyle \begin{array}{c}{v}^T Qv+{q}^Tv\\ {} subject\ to\\ {} Gv\preccurlyeq h\\ {} Av=b\end{array}} $$

for given *Q*, *q*, *G*, *h*, *A*, and *b*, where *Q* is a positive semidefinite (psd) matrix and the notation ≼ means the inequality is true for each coordinate (i.e., *y* ≼ *z* means *y*_*i*_ ≤ *z*_*i*_ for all *i*).

To put our optimization (2) in a QP formulation, we expand the covariance and variance terms in the definition of correlation in (1) and show that the covariance is *linear* in the transformation and variance is *quadratic*:

4 $$ Cov\left(w,{\rho}_l\right)={\left(\frac{1}{\mid P\mid }{a}_l-\frac{1}{{\left|P\right|}^2}{b}_l\right)}^Tw\kern1.5em and\kern1.25em Var(w)={w}^T\left(\frac{1}{\mid P\mid }S-\frac{1}{{\left|P\right|}^2}T\right)w $$

where *a*_*l*_ and *b*_*l*_ are *k*-dimensional vectors that depend only on D_1_ and *D*_*l*_; and *S* and *T* are *N* × *k* matrices that depend only on *D*_1_; and *P* is the set of pairs of observations, where |∙| denotes set cardinality. It is also not hard to show that $\frac{1}{\left|P\right|}S-\frac{1}{{\left|P\right|}^2}T$ is psd, as required. For details of the derivation, see the Additional file 1: Text S4.

There is one more difficulty to address. The correlation is the *quotient* of the covariance and the standard deviation, and the QP framework cannot handle quotients or square roots. However, maximizing a quotient can be reframed as maximizing the numerator (the covariance), minimizing the denominator (the variance), or both.

We now have the ingredients for the QP and can frame the optimization problem as

5 $$ {\displaystyle \begin{array}{c}\sum \limits_{j=2}^r{\gamma}_j\  Cov\left(w,{\rho}_j\right)-\alpha\ Var\left({\rho}^{\ast}\right)-\lambda {\left\Vert w-1\right\Vert}^2\\ {} subject\ to:\\ {} Cov\left(w,{\rho}_1\right)\ge \beta \\ {}w\succcurlyeq 0\end{array}} $$

where 0 and 1 are the all-zeros and all-ones vectors (of the appropriate length) respectively. Here, *λ* is the hyperparameter for regularization of *w*, which we want to penalize for being too far away from the all-ones vector (i.e., equal weighting of all the features). One could also regularize the *l*_2_ norm of *w* alone (i.e., incorporate the term −*λ*‖*w*‖^2^), which would encourage *w* to be small; we have found that empirically the choices yield similar results.

This program can be solved by standard QP solvers (see Additional file 1: Text S4, for the full details of how to put the above program in canonical form for a solver), and the solution *w*^∗^ can be used to transform unseen input data, using *u*^∗^ ∈ *R*^*k*^, where ${u}_i^{\ast }=\sqrt{w_i^{\ast }}$.

### Hyperparameters

A well-known challenge for machine learning algorithms is interpretability of hyperparameters. Here, the QP solver needs values for *λ*, *α*, and *β*, and specifying these in a principled way is a challenge for users. Our approach is thus to allow the user to specify more natural parameters. Specifically, we allow the user to specify minimum correlations between the pairwise distances in *D*^∗^ and the primary dataset *D*_1_. Formally, the user can specify *s* such that

$$ Corr\left({\rho}^{\ast },{\rho}_1\right)\ge s $$

and *q* such that

$$ \frac{\mathit{\max}\left\{w\right\}}{\sum \left|{w}_i\right|}=\frac{{\left\Vert w\right\Vert}_{\infty }}{{\left\Vert w\right\Vert}_1}\le \frac{q}{k}. $$

The quantity *q* thus controls the maximum weight that any one feature can take.

While these quantities are not directly optimizable in our QP formulation (5), we can access them by varying the hyperparameters *λ*, *α*, and *β*.

Intuitively, we note that the choice of *λ* controls whether *w* satisfies *q* and that *α* and *β* control whether the correlation constraint *s* is satisfied. To satisfy these constraints, we simply grid search across feasible values of {*λ*, *α*, *β*}: we solve the QP for fixed values of *λ*, *α*, and *β*, keeping only the solutions for which the {*s*, *q*} constraints are satisfied. Of these, we choose the most optimal. The efficiency of quadratic programming means that such a grid search is feasible, which gives users the benefit of more easily interpretable and natural hyperparameters.

### Recommendations for setting *s* and *q*

We recommend that only *s* (minimum correlation) and not *q* (maximum feature weight) be used to control Schema’s optimization. The default value of *q* in our implementation is set to be very high (10^3^) so that it is not a binding constraint in most cases. We recommend not changing it and in future versions of Schema we may reformulate the QP so that *q* is entirely removed. To limit the distortions in the primary modality, we recommend that *s* be set close to 1: the default setting of *s* is 0.99 and we recommend values ≥ 0.9. When Schema is used for feature selection, we recommend aggregating results across an ensemble of runs over a range of *s* values (a wide range is recommended here) to increase the robustness of the results.

### Preprocessing transforms

Standard linear decompositions, like PCA or NMF, are useful as preprocessing steps for Schema. PCA is a good choice in this regard because it decomposes along directions of high variance; NMF is slower but has the advantage that it is designed for data that is non-negative (e.g., transcript counts). Since the transform *u* that we generate can be interpreted as a feature-weighting mechanism, we can identify the directions (in PCA) or factors (in NMF) most relevant to aligning the datasets. Here the user can employ arbitrary feature sets including, for instance, a union of features from two standard methods (e.g., set-union of PCA and CCA features) or those generated by another single-cell analysis method, like MOFA+ ^21^.

### Motivating the choice of correlation as an objective

As a measure of the alignment between our transformation and a dataset, correlation of pairwise distances is a flexible and robust measure. Given a pair of datasets, the connection between their pairwise-distance Spearman rank correlation and the neighborhood structure similarity is deep: if the correlation is greater than 1 − *ϵ*, the fraction of misaligned neighborhood relationships will be less than $O\left(\sqrt{\epsilon}\right)$. There is a *manifold* interpretation that is also compelling: assuming the high-dimensional data lies on a low-dimensional manifold, small Euclidean distances are more accurate than large distances, so the *local* neighborhood structure is worth preserving. We can show intuitively that optimizing the correlation aims to preserve local neighborhood structure. Using correlation in the objective also affords the flexibility to broaden *Corr*(*w*, *ρ*_*j*_) in (2) to any function *f*_*j*_ of the metric, i.e., *Corr*(*w*, *f*_*j*_ ∘ *ρ*_*j*_); this allows us to invert the direction of alignment or more heavily weigh local distances. As RNA-seq dataset sizes reach millions of cells, even calculating the *O*(*N*^2^) pairwise distances becomes infeasible. In this case, we sample a subset of the pairwise distances. As an estimator, sample correlation is a robust measure, allowing Schema to perform well even with relatively small subsets; in fact, we only need a sample size *logarithmic* in our desired confidence level to generate high-confidence results (Additional file 1: Text S5). This enables Schema to continue scaling to more massive RNA-seq datasets.

### Inference of cell types by synthesizing gene expression and chromatin accessibility

Applying the *TruncatedSVD* function in the Python library *scikit-learn* [52] (version 0.23.1), we reduced the dimensionality of the primary (RNA-seq) and secondary (ATAC-seq) datasets to their top 100 and 50 components, respectively, and specified these as the inputs to Schema. We chose to perform SVD instead of PCA since only the former can work with sparse matrices (in particular, the ATAC-seq matrix had 11,296 rows and 247,293 columns). The minimum correlation threshold in Schema was set to 0.99 and Leiden clustering was performed with the Python package *leidenalg* [53] (version 0.8.1) with partitioning of the neighbor graph based on the modularity measure.

We performed canonical correlation analysis (CCA) on the same dimensionality-reduced primary and secondary datasets as supplied to Schema and computed 30 CCA factors, performing Leiden clustering using these.

We performed scVI and totalVI analysis using the Python package *scvi-tools* (version 0.8.1). To accommodate ATAC-seq data as an input to scVI and totalVI, we reduced the data’s dimensionality from 247,293 to 2629 by first excluding peaks with non-zero counts in fewer than 10 cells and then aggregating the count data of ATAC-seq peaks within 1 Mb genomic windows. To investigate if totalVI’s performance suffered because of the noise and sparsity in the ATAC-seq data, we evaluated it also on a synthetic, less-noisy dataset constructed by reusing and partially randomizing RNA-seq observations, our goal being to design a secondary modality that is not identical to the primary RNA-seq modality but nevertheless agrees well with it. We constructed each column of this dataset as the sum of the RNA-seq counts of 5 randomly chosen genes, with 10% of the final counts randomly set to zero. We found that totalVI did achieve stronger results when synthesizing this dataset with the RNA-seq modality (ARI of 0.088), substantially higher than what was achieved with using ATAC-seq as the secondary modality.

#### Pseudocells

We also evaluated a heuristic approach described in the original study [6]: group cells into small clusters (“pseudocells”) by RNA-seq similarity and compute an average ATAC-seq profile per pseudocell, using these profiles for the final clustering. This approach also underperformed Schema (ARI of 0.20). To implement the heuristic approach described by Cao et al. [6], we grouped the 11,296 cells into *k* = 300 clusters by k-means clustering of RNA-seq data; results were robust to the choice of *k*. Each cluster (“pseudocell”) was represented by the average ATAC-seq profile of its member cells, with these aggregated profiles forming the input to the Leiden clustering algorithm.

#### Schema highlights secondary patterns while preserving primary structure

We chose gene expression as the primary modality, reducing it with non-negative matrix factorization (NMF) to the top 50 components, and used temporal metadata as the secondary modality. We estimated differential pseudotime using the implementation in Scanpy [54] of Haghverdi et al.’s [55] algorithm.

#### Spatially informed differential expression on mouse brain Slide-seq

We used gene expression as the primary modality, while spatial density and cell type labels were the secondary modalities. We first computed spatial density information for each cell by learning a two-dimensional Gaussian-kernel density function on cell locations; it assigns higher scores to regions with denser cell packing (Fig. 4c). We then ran Schema using the gene expression matrix as the primary dataset, with the secondary datasets consisting of the numeric kernel density scores, as well as the categorical labels corresponding to the four most common non-granule cell types. We aimed to find a transformation of the primary data that maximized correlation with cell spatial density while preserving a high correlation with granule cell type labels. Additionally, differences in cell-type distribution between dense and sparse regions are a confounding factor when seeking to identify a gene set specific to the granule cell type. To mitigate this, we assigned a small negative weight to correlation with non-granule cell type labels in Schema’s objective function. The primary dataset was preprocessed with a non-negative matrix factorization (NMF) transformation, limiting it to the top 100 NMF factors. Each Schema run consisted of multiple sub-runs over an ensemble of parameter settings, with the results averaged across these. The gene scores from each sub-run were a weighted average of the features with each feature’s weight as *e*^*w*^, *w* being the Schema-computed weights; cell loadings were computed similarly. This softmax approach is parameter-free and ensures that gene rankings are informed primarily by the features with the highest Schema weight.

To adapt CCA for a three-way modality synthesis, we tested two approaches: (1) combining spatial density and cell-type information into a composite measure that was then correlated to gene expression, or (2) performing two separate CCA analyses (correlating gene expression against either spatial density or cell type) and combining them. In the first CCA-based approach, we combined spatial density and cell-type labels by learning a Gaussian kernel density function only on cells labeled as granule cells and then inferring its value for other cells. This score was then used in CCA. In the second CCA-based approach, where we integrated results from two preliminary CCA runs, the combined cell loadings were computed as the average of the normalized cell loadings from the two CCAs, with the final gene scores then computed by a matching pursuit technique [56, 57]: the final CCA score of a gene was the dot product of the CCA cell loadings and the gene’s expression vector. In our evaluations, the first CCA-based approach performed comparably or worse than the second, and the results for only the latter are presented in this paper.

We also needed to adapt SpatialDE and Trendsceek, both of which have unsupervised formulations, to select for genes whose expression shows spatial variation in granule cell types but not in non-granule cell types. To do so, we ran them separately on granule and non-granule cells and then ranked genes based on the difference of gene ranks between the two runs.

#### Schema reveals CDR3 segments crucial to T cell receptor binding specificity

When estimating location-specific selection pressure (Fig. 5c, d), we truncated CDR3 sequences to the first 20 residues (sequences longer than that constituted less than 0.2% of our dataset). The *i*th element of the primary modality feature vector was the 1-letter code of the amino acid at the *i*th sequence position or a null value if the sequence length was shorter than *i*. We defined the distance between two sequences as the number of elements that disagreed. In the original space, this corresponds to the Hamming distance; in the transformed space, it is a location-weighted version of the Hamming distance. The secondary modality corresponding to binding specificity against the 44 ligands was represented as a 44-dimensional Boolean vector, with the Euclidean distance metric. Each Schema run was an ensemble of sub-runs, with varying parameter choices of minimum correlation between the original and transformed datasets and the maximum allowed feature weights. Feature weights produced in each sub-run were normalized by linearly mapping the lowest weight to 0 and the highest to 1.

We then averaged these normalized feature weights across sub-runs. To compute a location’s score using VDJdb, we extracted the VDJdb-provided relative entropy score (*I.norm*) for the location in each TCR motif and averaged it across all motifs in the database. Here, Schema and VDJdb scores have opposite orientations: for a location that demonstrates low variability, the associated Schema weight will be high while the VDJdb score will be low. Therefore, when comparing the Schema and VDJdb scores, we inverted the orientation of Schema scores by subtracting them from 1 (Fig. 5c, d).

The comparison of per-location scores between Schema and VDJdb is complicated by length differences between motifs in VDJdb and sequences in our dataset: the former contains shorter sequences, with the average sequence length of α and β chain motifs in VDJdb being 11.9 and 12.5, respectively; the corresponding averages in our dataset are 13.5 and 14.5. However, both datasets have good coverage of locations 1–10 and the per-location scores are in broad agreement there (Fig. 5c, d).

To compute the selection pressure on amino acids, we focused on segments 3–7 in TCR α chains and 5–11 in TCR β chains, choosing these locations for their high sequence variability as estimated by Schema and VDJdb above. To compute Schema scores, an ensemble of sub-runs was performed, and as described above, Schema scores were normalized. VDJdb scores for an amino acid were computed as the average frequency-weighted relative entropy scores (*height.I.norm*) across the selected locations in all TCR motifs in the database.

To exemplify how Schema can synthesize additional modalities, we also incorporated proteomic measurements of 12 cell-surface markers. Hypothesizing that cell-surface protein levels should be unrelated to the V(D)J recombination variability, we added a low weight term to Schema’s objective function that *penalized* correlation between distances in the CDR3-sequence space and distances in proteomic-measurement space. Across subsets of the dataset split by donors (4 subsets) or by epitopes (10 randomly divided subsets), we compared the baseline two-modality setup against the new three-modality setup and found that the latter produced slightly more stable results than the former, with smaller standard deviations of Schema-computed weights across the subsets of data (0.094 vs 0.101 for the donor split, and 0.164 vs 0.166 for the epitope split). In general, we recommend the use of cross-validation or an independent metric to calibrate the relative weights of secondary modalities in such use-cases.

### Supplementary Information

**Additional file 1:.** Figures S1-S10, Text S1-S6, Table S1.

**Additional file 2:.** Tables S2, S3.

## Data Availability

Python source code [58] of Schema, under the open source MIT license, is available at https://github.com/rs239/schema . Its documentation and example usage is available at https://schema-multimodal.readthedocs.io/. The program is also available as the Python package “*schema_learn*” that can be installed using the Python package installer, pip. The version of software used in this manuscript has been deposited in Zenodo [59]: 10.5281/zenodo.4521803. We used the following publicly available datasets. Below, GEO refers to the Gene Expression Omnibus repository (https://www.ncbi.nlm.nih.gov/geo/): • Slide-seq data from Rodriques et al. [10] ° https://singlecell.broadinstitute.org/single_cell/study/SCP354/slide-seq-study ° Processing code at: https://github.com/broadchenf/Slideseq/tree/master/BeadSeq%20Code • Sci-CAR (ATAC-seq and RNA-seq) data from Cao et al. [6] (GSE117089 from GEO). • Topologically Associating Domains data for A549 cells from ENCODE (accession ENCFF336WPU) [60, 61]. • Multimodal T cell receptor data from 10x Genomics [5]. ° https://www.10xgenomics.com/resources/datasets/#dataset-accordion-221-3-0-2-content • T cell motif data from Shugay et al.’s [43] VDJdb database: ° Website: https://vdjdb.cdr3.net/ ° Bulk data (contains motif_pwms.txt, a file describing position-weight matrices of motifs): https://github.com/antigenomics/vdjdb-db/releases/tag/2020-01-20 ° Older version of the VDJdb motif data (does not incorporate the 10x Genomics multimodel TCR data): https://raw.githubusercontent.com/antigenomics/vdjdb-motifs/master/motif_pwms.txt • Davie et al.’s [3] RNA-seq data of the aging *Drosophila* brain (GSE107451 from GEO). • Argelaguet et al.’s [22, 46] preprocessed version of Pjiuan-Sala et al.’s [45] RNA-seq data on mouse gastrulation and their pretrained models, available at ftp://ftp.ebi.ac.uk/pub/databases/mofa/scrna_gastrulation/ • Hochgerner et al’s [47], RNA-seq data on dentate gyrus neurogenesis (GSE95753 from GEO), made available as a sample dataset in the Python package *scvelo*. We used the following software packages: • Python (version 3.6.1): *scanpy* (version 1.5.1), *scikit-learn* (version 0.21.3), *scvelo* (version 0.2.1), *pandas* (version 0.25.1), *numpy* (version 1.17.1), *scipy* (version 1.5.1), *SpatialDE* (version 1.1.3), *leidenalg* (version 0.8.1), *scvi-tools* (version 0.8.1). • R (version 3.6.3): *MOFA2* (version 1.1), *trendsceek* (https://github.com/edsgard/trendsceek).
