# Supplementary material for: Schema: metric learning enables interpretable synthesis of heterogeneous single-cell modalities
Source: Genome Biol. 2021 May 3;22:131. doi: 10.1186/s13059-021-02313-2 (PMC8091541; doi:10.1186/s13059-021-02313-2)
Supplement: Supplementary file 1 — Additional file 1:. Figures S1-S10, Text S1-S6, Table S1. [file 13059_2021_2313_MOESM1_ESM.docx]

**Supplementary Materials**

for

**Schema: metric learning enables interpretable synthesis of heterogeneous single-cell modalities**

Figures S1-10

Text S1-6

Table S1

(Tables S2-3 are included in Additional file 2)

**Figure S1**

**Additional Demonstration: Batch-effect adjusted identification of differentially expressed genes along a developmental time course. (See Text S1 for details)**


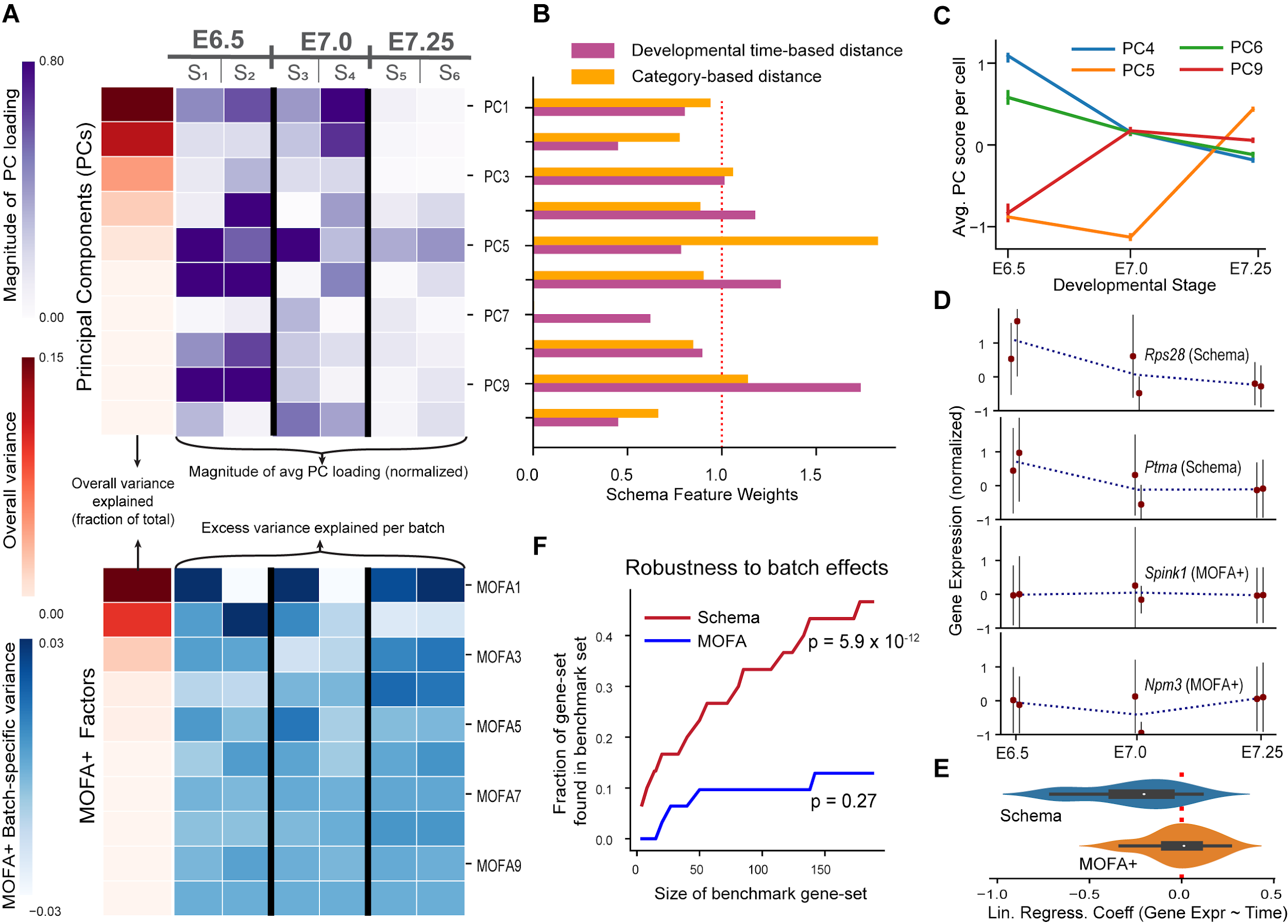


(**A**) We obtained a dataset of developing mouse epiblast cells spanning three timepoints, with two experimental batches per timepoint. PCA and MOFA+ components show significant within-timepoint variability. In this panel, loadings of each principal component (PC) were normalized to zero mean and unit standard deviation. (**B, C**) Weights computed by Schema after accounting for batch effects and developmental age with two different distance metrics, one that provides Schema with temporal-ordering and another that does not provide this order. When incorporating order information, Schema down-weights PC5, which shows substantial within-timepoint, batch-related variability, and up-weights PC9, which has higher correlation with time. Correspondingly identified PCs reflect the effect of these metric. (**D, E**) Schema identifies genes with monotonically changing expression. For each gene identified by Schema or MOFA+, we regressed its expression (normalized to zero mean and unit standard deviation) against developmental time, encoding stages E6.25, E7.0 and E7.25 as timepoints 1, 2 and 3, respectively. Consistent with stage-dependent monotonicity in expression, the fitted slopes for Schema genes were significantly different from zero (two-sided *t*-test, *p* = 3.83 x 10^-6^); this was not true of MOFA+ (*p* = 0.77). (**F**) Schema has stronger overlap with batch-effect adjusted benchmark sets of differentially expressed genes (hypergeometric test with Bonferroni correction, *p* = 5.9 x 10^-12^ for the benchmark set of size 188).

**Figure S2**

**Additional Demonstration: Synthesis of spliced and unspliced mRNA counts recovers RNA velocity and enables informative visualization. (See Text S2 for details)**

**
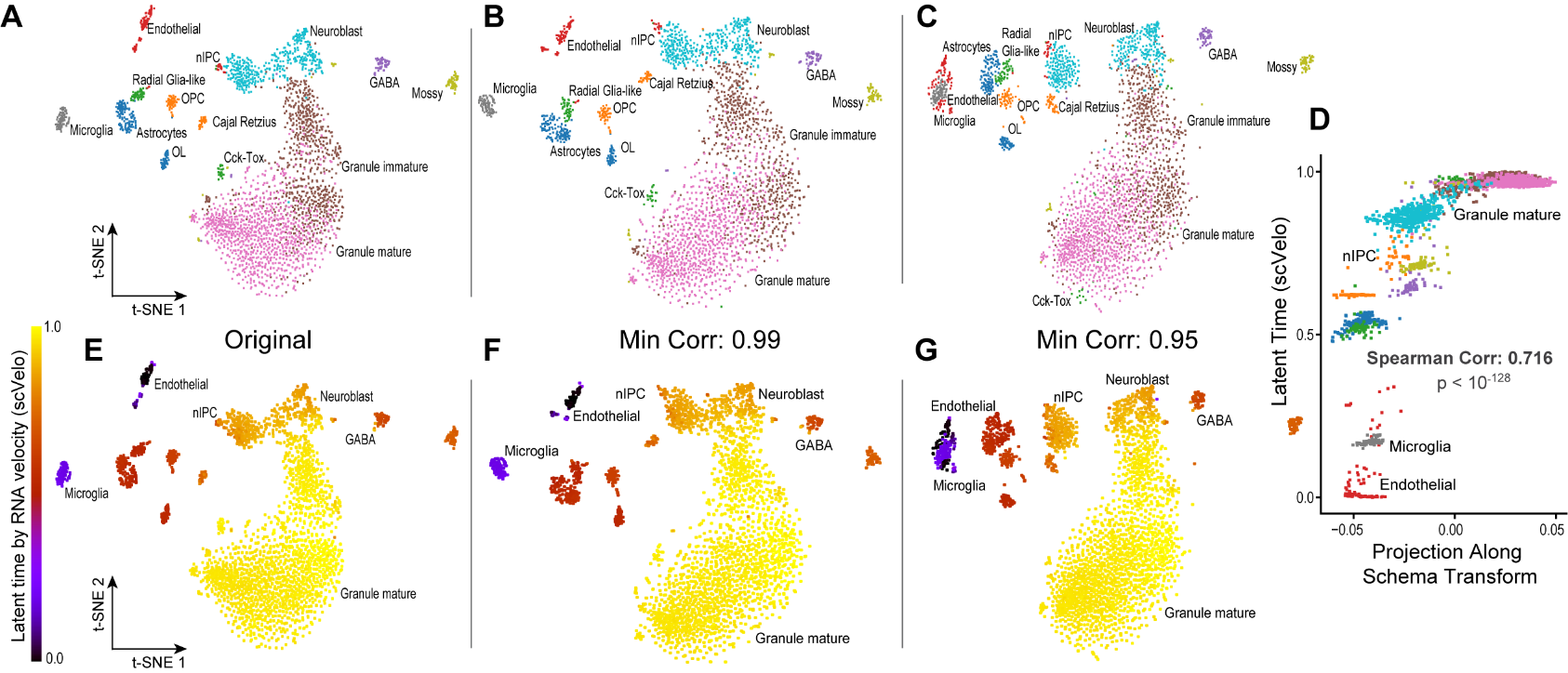
**

(**A**): t-SNE visualization of the spliced mRNA counts (**B-C**): We synthesized spliced and unspliced mRNA counts, with the former as the primary and the latter as the secondary modality, respectively. Schema’s transformation picks up the time derivative of gene expression, thus accentuating the cell differentiation process. t-SNE visualizations of synthesized data with 0.99 and 0.95 minimum correlation, respectively, are shown. (**D**) Schema’s results are in agreement with the RNA velocity tool, scVelo. By measuring each cell’s Schema transformation, we computed a pseudotime estimate which we found to be significantly correlated with scVelo’s latent-time estimate (Spearman rank correlation = 0.716, two-sided t-test *p* < 10^-128^). (**E-G**): Same t-SNE visualizations as above, but with cells colored by their scVelo latent-time, showing that Schema puts cells at similar differentiation stages progressively closer.

**Figure S3**

1. **Leiden clustering for RNA-seq and ATAC-seq data individually and Schema’s synthesis**


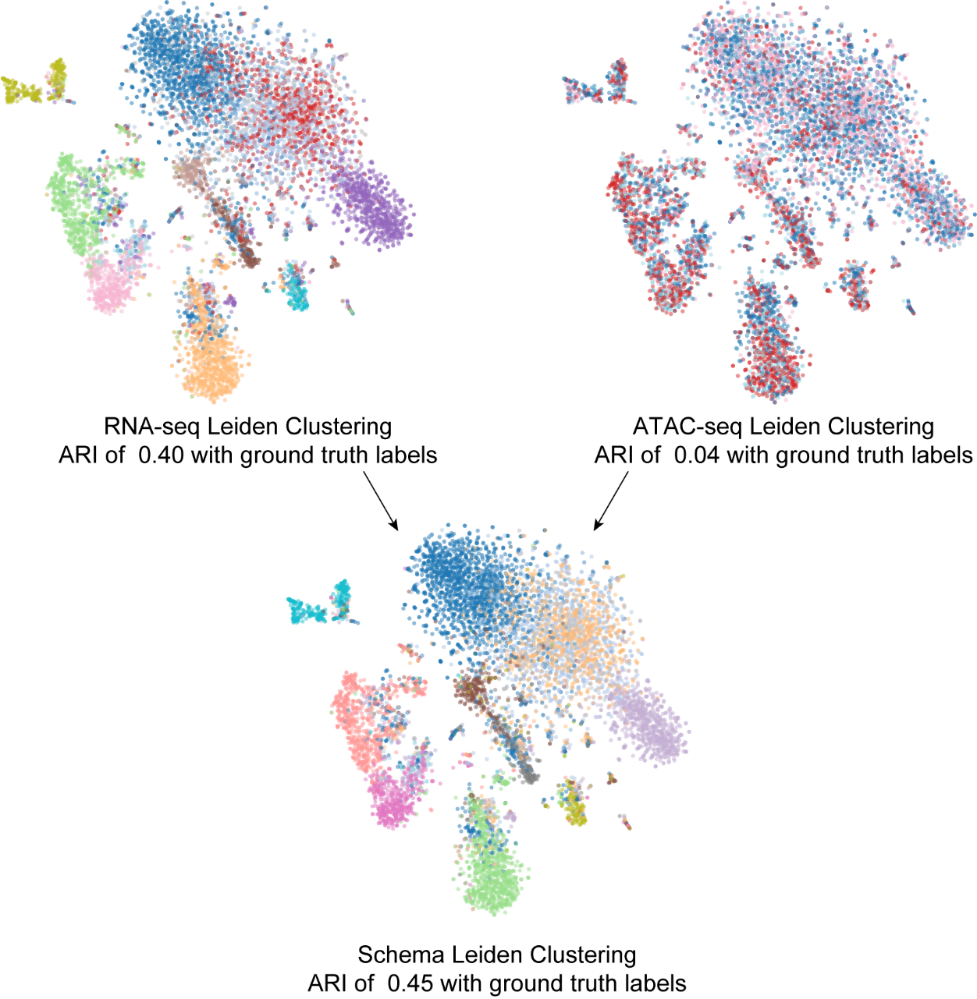


1. **Leiden clustering for totalVI and CCA synthesis**


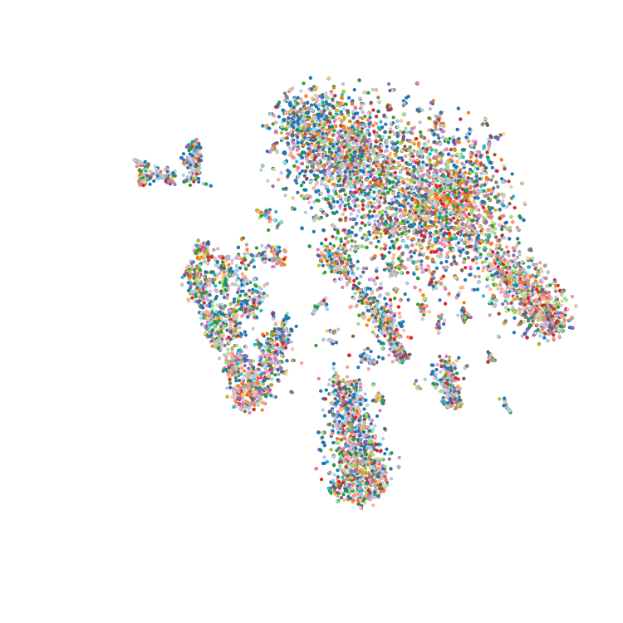

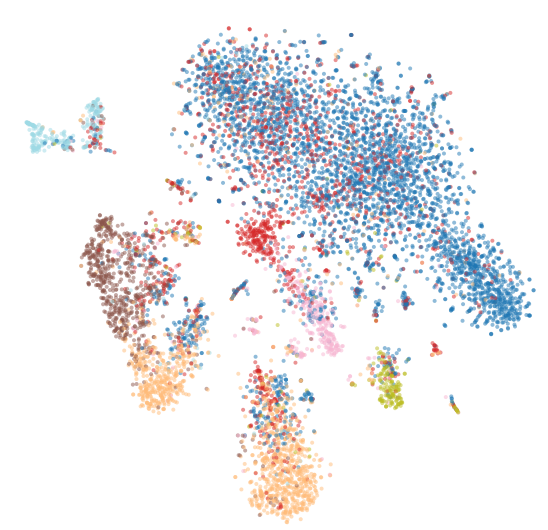


**Figure S4**

**Comparison of Schema with CCA on Slide-seq data (Part 1)**


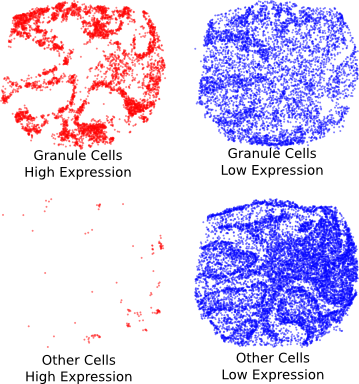


Visually, CCA seems to be effective at identifying a gene set that is differentially expressed only in densely-located granule neurons. The Slide-seq sample used here (Puck 180430_1) is the same as in **Fig. 4D**. However, as shown in **Fig. 4E**, the gene ranking computed by Schema is better preserved across three Slide-Seq samples than those produced by CCA (median sample-pair Spearman rank correlation of 0.675 and 0.457, respectively)

**Figure S5**

**Comparison of Schema with CCA on Slide-seq data (Part 2)**


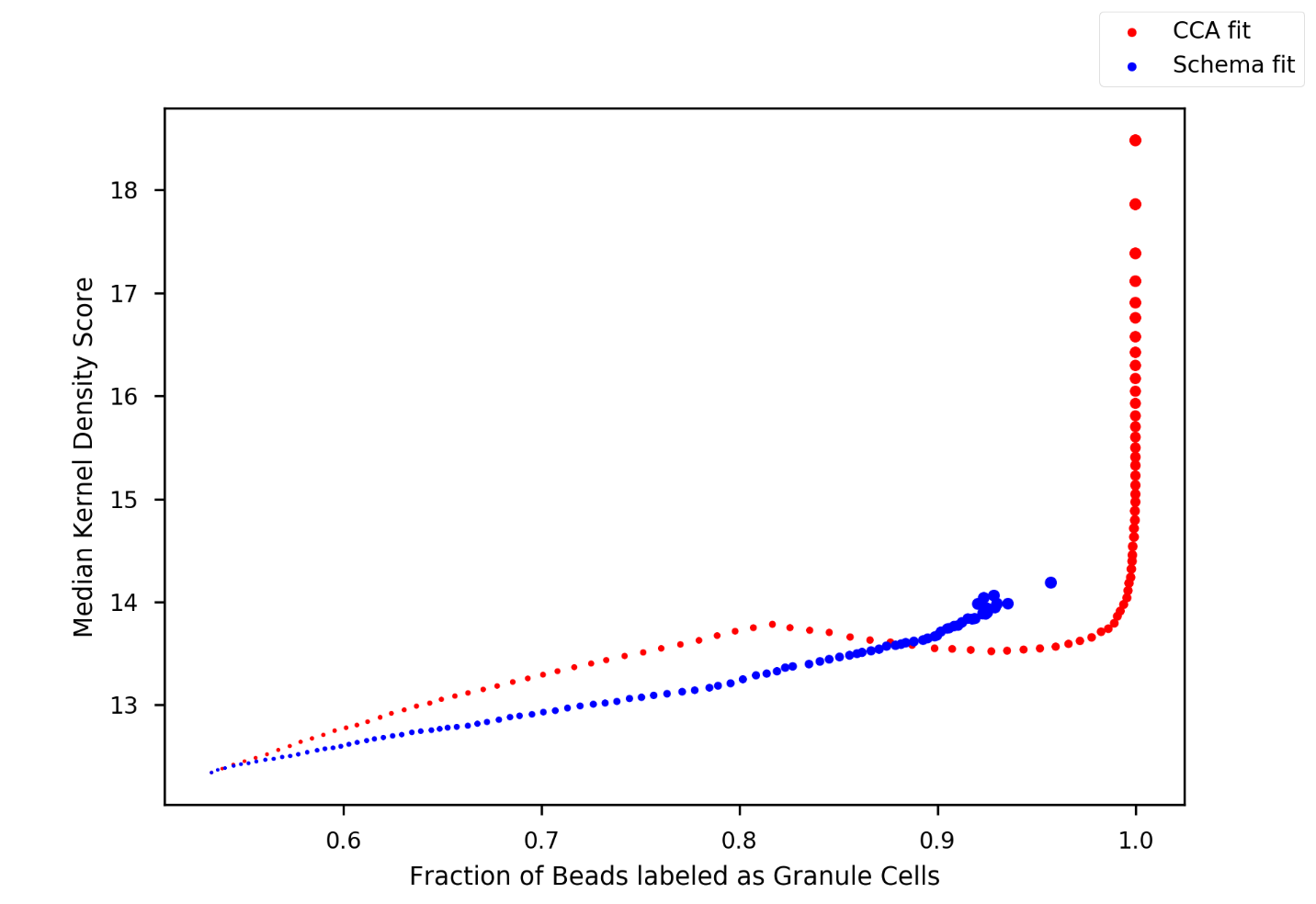


*Investigation of CCA and Schema cell loadings*: We sorted Slideseq transcriptomes by their loading on the Schema-implied gene scores and investigated how the exposure to secondary modalities (cell-type labels and spatial density) varied in this ordering; we repeated the analysis for CCA cell loadings. For *k*=1,...,99, we selected cells with loadings in the percentile range *[k, 100]* and computed the frequency of granule-cell labels and the average Gaussian kernel density score of a cell in this set; higher values of these measures indicate stronger agreement with the cell type and spatial density modalities, respectively. In the plot, the size of a point is proportional to *k*. For both Schema and CCA, the higher cell loadings typically correlate with a higher granule-cell fraction and higher kernel density, as both the methods transform the primary gene-expression modality to align it with the secondary modalities. However, for Schema this relationship plateaus after a point because Schema’s regularization mechanism limits the distortion of the primary modality, constraining the extent of match with the secondary modalities. In contrast, the unconstrained framework of CCA produces loadings where the 99^th^ percentile cell loading has significantly higher spatial density exposure than the 95^th^ percentile. This may lead to overfitting as CCA computes gene rankings that are overly determined by sample-specific artifacts. In contrast, the regularization mechanism of Schema produces gene rankings that are better preserved across samples.

**Figure S6**

**Evaluation of canonical correlation analysis (CCA) performance (also see Fig. S4, S5)**


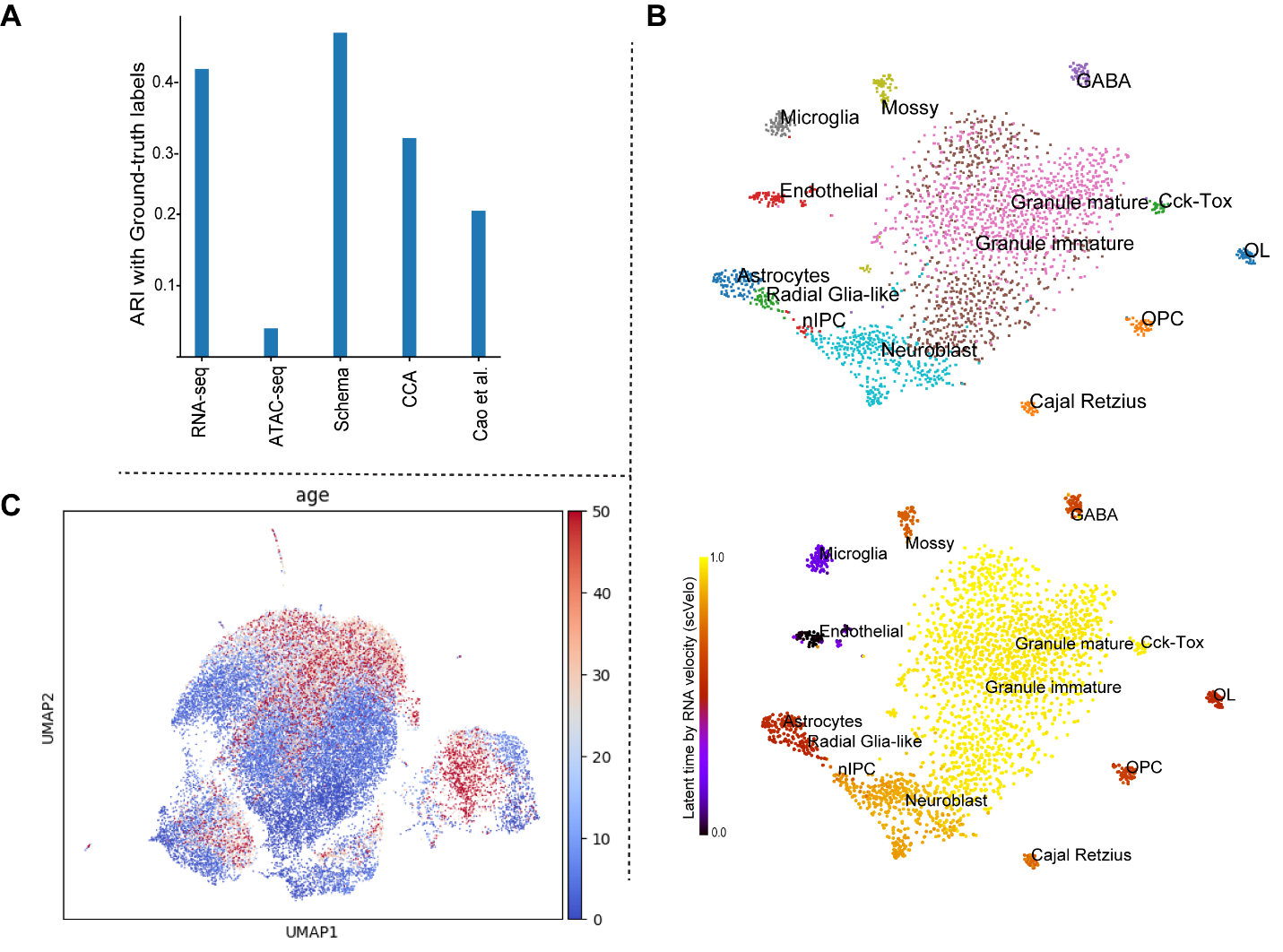


**(A)** *Inferring cell types by synthesizing RNA-seq and ATAC-seq data*. The metric of evaluation here is the agreement between Leiden clustering on the synthesized dataset and ground truth cell-type labels, measured using the adjusted Rand index (ARI), with higher scores indicating greater agreement. This panel contains the same information as **Fig. 2E** and is reproduced here for convenience. **(B)** *Inferring RNA velocity by synthesizing spliced and unspliced mRNA counts*. Spliced and unspliced data were correlated using CCA and the synthesized data was visualized with t-SNE. The bottom half of the panel colors cells by their scVelo latent-time estimate. This panel should be compared with **Fig. S2B-F**, which display the corresponding plots produced by Schema synthesis of the data. CCA’s synthesis does not place cells with similar stages of differentiation as closely together as Schema. Quantitatively, the Spearman rank correlation between t-SNE distances and latent-time difference is 0.163 for CCA, less than the correlation achieved using just the spliced mRNA counts (0.397); in contrast, the Schema transformation corresponding to a minimum correlation constraint of 0.95 results in a correlation of 0.432. **(C)** *Schema highlights secondary patterns while preserving primary structure.* RNA-seq data was synthesized with cell age metadata using CCA. Compared to a synthesis by Schema (**Fig. 3B-D**), the CCA-based visualization less clearly communicates the developmental trajectory. We quantified the age-related structure in the transformed dataset by a diffusion pseudotime analysis. The Spearman rank correlation between the pseudotime estimate and the ground-truth cell age is only 0.059 for the CCA-synthesized data while it 0.365 in the original, untransformed dataset and 0.436 in the Schema-transformed dataset corresponding to a minimum correlation constraint of 0.99.

**Figure S7**

**Correlation of factor loadings between MOFA+ factors and principal components (See Text S1)**


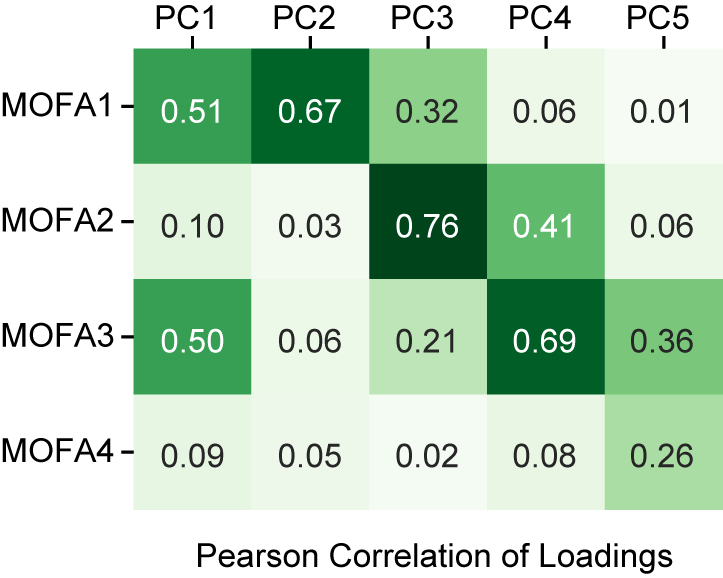


**Figure S8**

**Differential expression analysis while accounting for batch effects and developmental stage (See Text S1):** Schema feature-selection results for different weights of the developmental-stage and batch-effect modalities.

The middle column is the one shown in the main text: equal (and opposite) weights for the batch-effects and developmental-stage modalities. The left-most column corresponds to using only the batch-effect modality while the right-most column corresponds to using only the developmental-stage modality.


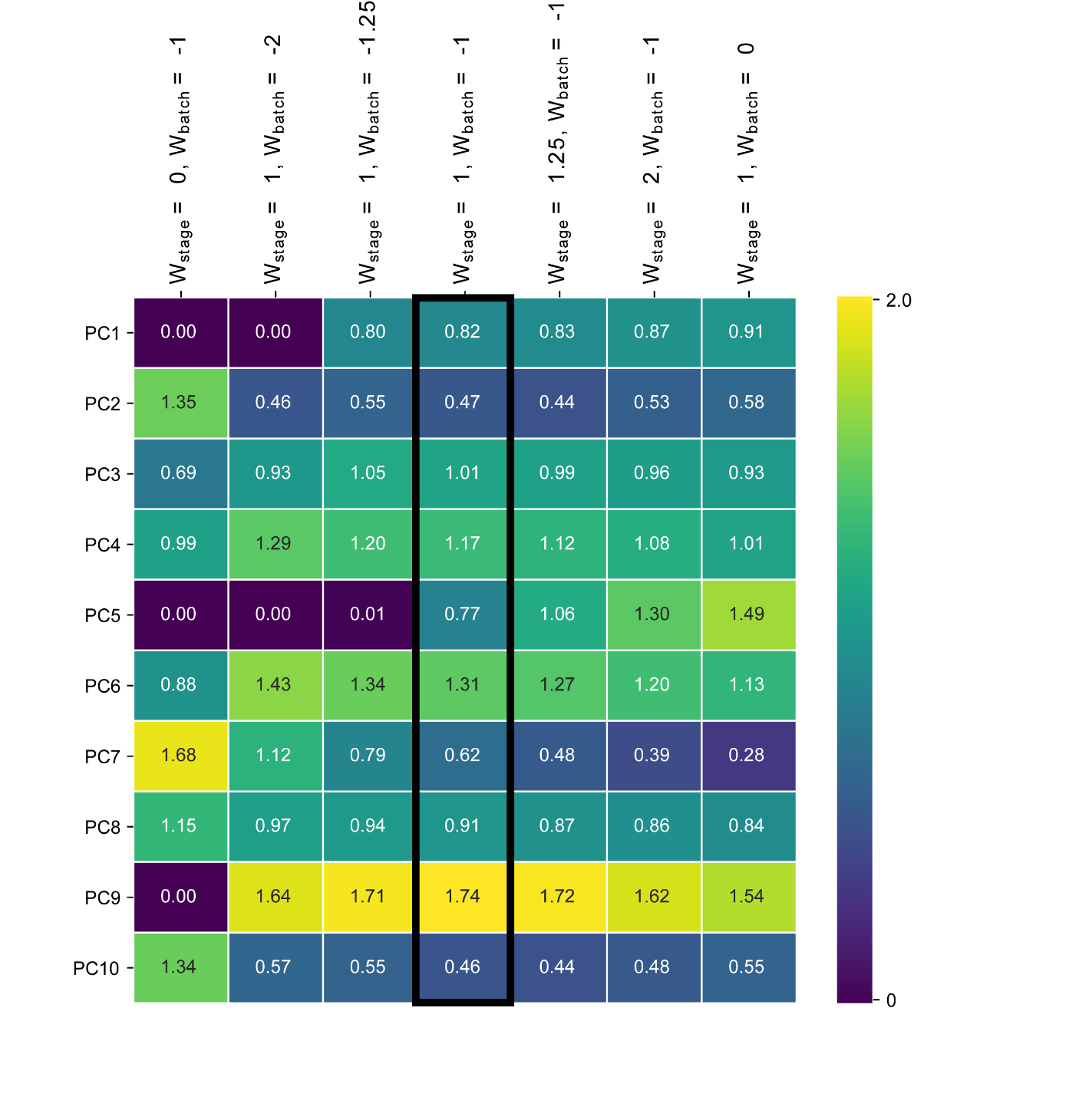


**Figure S9**

**Visualization of enriched GO terms in Schema-ranked genes across 3 samples of Slide-Seq data** (via REViGO^29^, using abs(log10(*p*-value)) )


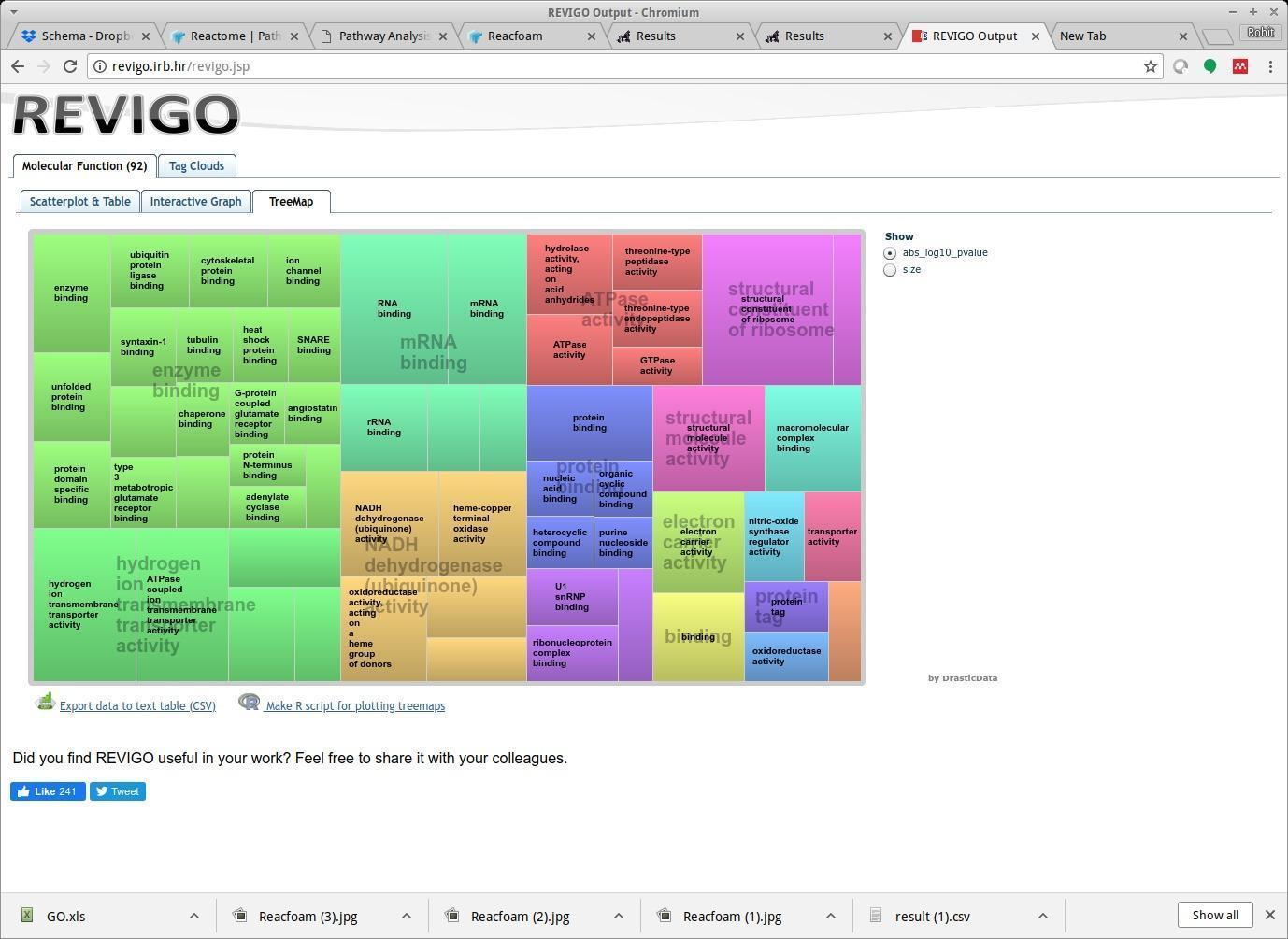


**Figure S10**

**Voronoi-tessellation visualization of REACTOME pathways enriched in Schema-ranked genes across 3 samples of Slide-Seq data**


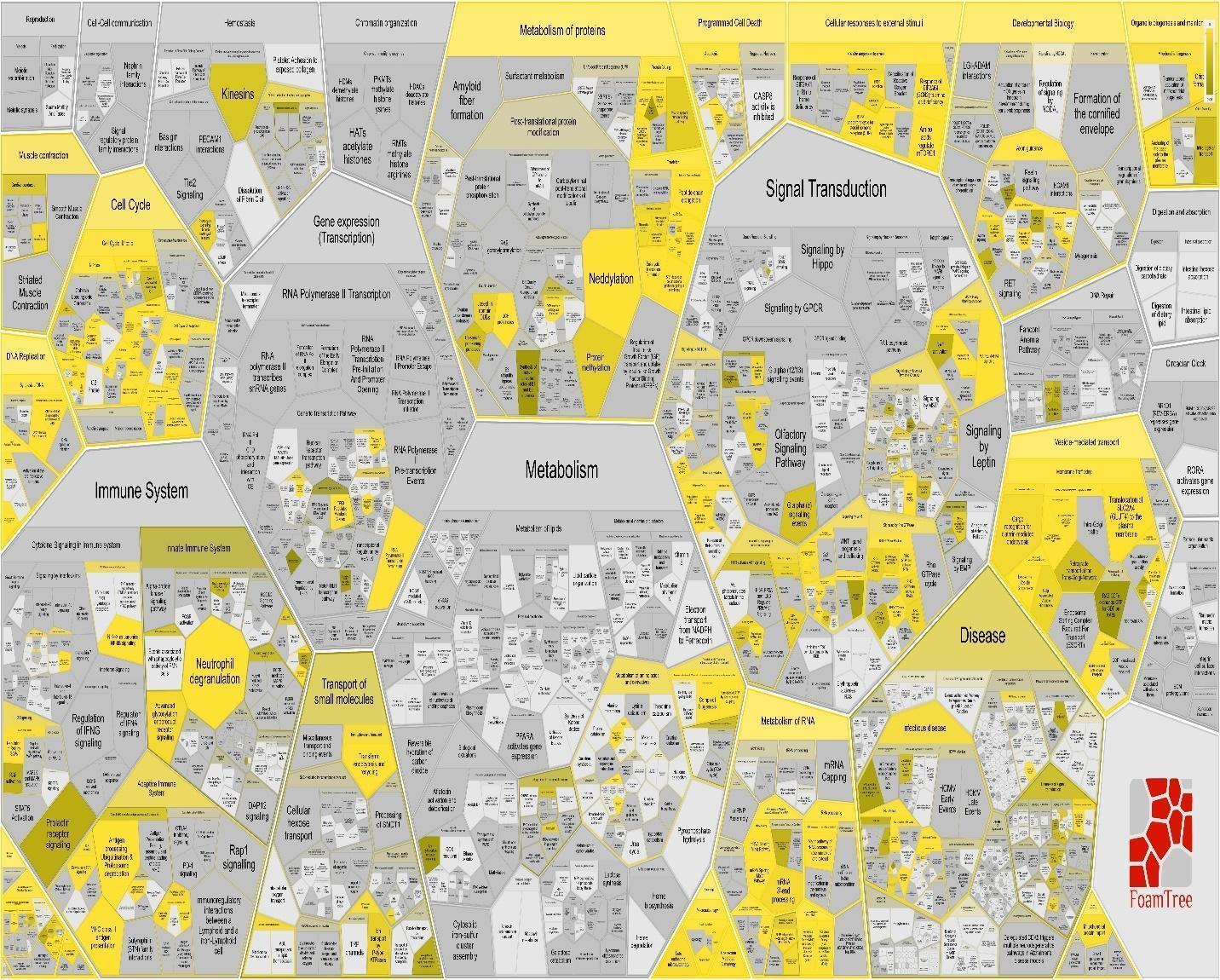


**Text S1**

**Additional Demonstration:** *Differential expression analysis while accounting for batch effects and developmental age*

Aside from cell type inference, another important single-cell analysis task that stands to benefit from multimodal synthesis is the identification of differentially expressed marker genes. To illustrate how, we explored a mouse gastrulation single-cell dataset [1], consisting of 16,152 epiblast cells split over three developmental timepoints (E6.5, E7.0, and E7.25) and with two replicates at each timepoint, resulting in six distinct batches (**Fig. S1A**). Applying Schema to this dataset, we sought to identify differentially expressed genes that are consistent with the developmental time course while being robust to batch effects between the replicate pairs. To perform differential expression analysis with Schema, RNA-seq data should be used as the primary modality, while the distance metrics of the secondary modalities specify how cells should be differentiated from each other. Here, we used batch and developmental-age information as secondary modalities, configuring Schema to maximize RNA-seq data’s agreement with developmental age and minimize its agreement with batch information. We weighted these co-objectives equally; results were robust to ± 25% variations in these weights (**Fig. S8**). We used RNA-seq data as the primary dataset, representing it by its top ten principal components. (**Methods** below).

We evaluated Schema alongside MOFA+ [2], a recently introduced single-cell multimodal analysis technique. Schema and MOFA+ approach the data synthesis problem from complementary perspectives: while the emphasis in Schema is to identify important features of the primary dataset and its corresponding transformation that reflects a synthesis of the various modalities, MOFA+ focuses on *de novo* identification of features that explain the covariation across modalities. In Argelaguet et al.’s MOFA+ analysis of this dataset, the authors identified 10 factors that capture similar information to the top principal components (**Fig. S7**). To identify differentially expressed genes with MOFA+, we selected the top genes from two factors (MOFA1 and MOFA4) reported by Argelaguet et al. as capturing developmental variation (**Methods** below).

In addition to accounting for batch effects, we could also configure Schema to reduce the weight of transient changes in expression, thus identifying genes with monotonically changing expression along the time course (**Fig. S1B-D**). To do so, we encoded developmental age as a distance metric by specifying zero distance between cells at the same timepoint, unit distance between directly adjacent timepoints, and an additive sum of the unit distances across more separated timepoints. As a control, we also tested a metric that did distinguish between the stages but did not increase in time, finding that the highest-weighted feature (PC5) in that case was indeed non-monotonic (**Methods** below, **Fig. S1B-C**). To encode batch effect as a distance metric, we specified zero distance between cells in the same replicate and unit distance otherwise.

We estimated the set of differentially expressed genes as the top-loading genes of the principal components up-weighted by Schema. Seeking to evaluate if the Schema or MOFA+ genes did show time-dependent monotonicity in expression, we linearly regressed each identified gene’s normalized expression against an ordering of the three developmental stages (**Methods** below). We found that the Schema genes corresponded to regression coefficients significantly different from zero (**Fig. S1D-E**), consistent with time-dependent monotonicity (two-sided *t*-test, *p* = 3.83 x 10^-6^); this was not true of MOFA+ (*p* = 0.77).

Next, we evaluated the batch-effect robustness of Schema and MOFA+ gene sets. Our configuration of Schema balances batch-effect considerations against differential expression considerations. For instance, introducing the batch-effect objective in Schema reduces the weights associated with the first and second principal components (PC1 and PC2), which show substantial within-timepoint batch-effect variations without a compensating time-dependent monotonicity, by 11% and 17%, respectively. In comparison, explicitly up-weighting "good" variation or down-weighting "bad" variation is difficult when using MOFA+. To systematically evaluate the batch-effect robustness of Schema and MOFA+ gene sets, we constructed benchmark sets of differentially expressed genes by applying a standard statistical test, adjusting for batch effects by exploiting the combinatorial structure of this dataset. Specifically, we aggregated over computations that each considered only one replicate per timepoint (**Methods** below). We then measured the overlap of Schema and MOFA+ gene sets with these benchmarks (**Fig. S1F)** and found that, compared to MOFA+, the Schema gene set shows a markedly higher overlap with the benchmarks that is statistically significant (hypergeometric test with Bonferroni correction, *p* = 5.9 x 10^-12^ for the benchmark set of size 188). Schema allows us to express the intuition that variation attributable to batch effects should be ignored while variation attributable to developmental age should be highlighted.

**Methods**

This mouse gastrulation dataset was originally described by Pijuan-Sala et al. [1] and investigated by Argelaguet et al. using the MOFA+ [2] algorithm. We operated on the data as preprocessed and made available by them and, for the MOFA+ evaluation in this paper, also used their pretrained models.

We first reduced the RNA-seq data (primary modality) to its top 10 principal components (PCs), to be in line with the 10 MOFA+ factors from Argelaguet et al. The MOFA+ algorithm can be thought of as a generalization of PCA and we did indeed observe that the top PCs were very similar to the top MOFA+ components (**Fig. S7**), validating that Schema was able to access the same sources of variation as found by MOFA+ here.

We configured Schema to use batch information as a secondary modality with weight -1 and developmental age information as a secondary modality with weight +1; thus, correlation with the former was minimized and the latter was maximized. The minimum correlation threshold was set to 0.9; we found that the results were robust to variations in this setting (0.8 and 0.95).

Since Schema accepts arbitrary distance measures on secondary datasets, we could investigate the impact of treating developmental timepoints as categories rather than a time ordering. In the categorical distance metric, we defined two cells to be at distance 0 if they were at the same developmental timepoint and at distance 1 otherwise. In the time-ordering metric, we specified the first and third time-points to be at distance 2 apart and the middle time-point to be at distance 1 from either end. The two distance measures lead to different feature-selection results from Schema, reflecting the distinct underlying variations in expression profiles. For category-based distances, PC5 receives the highest weight while PCs 4 and 6 are given higher weights in the time-ordering case (**Fig. S1B**). This happens because the mean expression level of PC5 shows large variation across the three time-points but does not change monotonically along the time course; in contrast, the PCs 4 and 6 display expression profiles that change monotonically with developmental age (**Fig. S1C**). Schema’s flexibility to incorporate a distance measure that highlights specific variability patterns can thus enable researchers to identify precisely targeted gene-sets.

To create a gene set from Schema’s feature weighting, we selected the intersection of *k* top loadings (by absolute value) of PCs up-weighted by Schema (PCs 4, 6 and 9 for the time-ordering metric); we choose *k* so that the intersection contained 30 genes. For MOFA+, we chose genes that had the top loadings (by absolute value) in the factors MOFA1 or MOFA4. Here, we were following Argelaguet et al who, after an investigation of the various MOFA+ factors, had identified these two as the most relevant to understanding developmental age variability. Since the top loadings of the two MOFA+ factors do not overlap much, we chose the top 16 genes from each, with their union consisting of 31 genes (there was one overlap between the two subsets).

For each gene identified by Schema or MOFA+, we regressed its expression against developmental time, encoding stages E6.25, E7.0 and E7.25 as timepoints 1, 2 and 3, respectively. The gene’s expression profile (across all cells) was first normalized to zero mean and unit standard deviation.

We created batch-effect adjusted benchmark gene sets by using different combinations of replicates. One can create a subset of the original dataset by sampling cells from only one of the two replicates at each time-point. By iterating over all possible combinations of replicates, we created eight such subsets. These subsets differ in the batch information they contain but share the same developmental age information. Using the Wilcoxon rank sum test in *scanpy*, we identified genes differentially expressed between the first (E6.5) and last (E7.25) stage in each subset and defined the benchmark gene set to consist of genes that are differentially expressed across a majority of the subsets. The benchmark set is thus robust to batch effects, being comprised of genes whose differential expression stands out across different replicates (i.e., batches). By varying the thresholds of the test, we could create benchmark sets of varying sizes and measured the overlap of Schema and MOFA+ gene sets with these. The Schema gene set has a higher overlap and for benchmark sets of all sizes, its overlap with them was significant (hypergeometric test with Bonferroni correction, *p* = 5.9 x 10^-12^ for the benchmark set of size 188 and Bonferroni-corrected *p* < 10^-5^ for benchmark sets of all sizes, **Fig. S1E**); this was not the case for MOFA+.

**Text S2**

**Additional Demonstration:** *Schema can synthesize spliced and unspliced RNA counts to accentuate cell differentiation*

We next leveraged the flexibility of Schema to study cell differentiation by synthesizing spliced and unspliced mRNA counts in a dataset of 2,930 mouse dentate gyrus cells [3]. Specifying spliced counts as the primary dataset and unspliced counts as the secondary dataset, we configured Schema to compute a transformation of the spliced data that maximizes the correlation of its Euclidean distances with those in the unspliced dataset while distorting the former only minimally (**Fig. S2A-C**), (**Methods** below)

Our intuition here is the same as that underlying RNA velocity techniques: correlating spliced and unspliced counts in a cell should pick up on the time derivative of a cell's expression state and thus illuminate the cell differentiation process. To validate this intuition, we computed a pseudotime measure from the difference between transformed and original RNA-seq data, finding it to be highly correlated with the latent-time estimate produced by Bergen et al.’s RNA velocity tool scVelo [4] (Spearman rank correlation 0.72, two-sided *t*-test *p* < 10^-128^, **Fig. S2D**, **Methods** below). Since Schema relies on the same underlying biological phenomena as specialized RNA velocity tools but analyzes the data differently, these results show the breadth of Schema’s generality and may be used to help supplement and strengthen the findings from standard RNA velocity analyses.

Schema can complement methods like scVelo by facilitating additional analyses. As in our demonstrations of cell type inference and UMAP visualization, the transformed data produced here by Schema incorporates additional information (the time derivative of expression) but remains analyzable as an RNA-seq dataset. As an example, we visualized the transformed dataset with t-SNE, finding that the two-dimensional t-SNE plot of the Schema-transformed data places more closely together cell types at similar stages of differentiation (as quantified by scVelo latent-time, **Fig. S2E-G**). To confirm this visual observation, we computed the Spearman rank correlation of scVelo latent-time differences between pairs of cells and their corresponding Euclidean distances in the t-SNE embedding space, finding that it increases from 0.397 in the original dataset to 0.432 in the transformation corresponding to a minimum correlation constraint of 0.95 (**Methods** below). In contrast, an unconstrained synthesis using CCA produced a substantially lower correlation of 0.163; see **Fig. S6** for the corresponding CCA-based t-SNE visualization. Schema can thus facilitate visualizations that reflect the deeper underlying differentiation processes.

**Methods**

We normalized the spliced and unspliced counts, log transformed them and reduced them to their top 100 principal components. These were specified to Schema as the primary and secondary modalities, respectively.

To construct a pseudotime estimate from Schema’s output, we first computed the mean per-cell difference between the transformed and original RNA-seq data. Interpreting this difference as the major axis of transcriptional change, we projected the original RNA-seq values on it. The magnitude of projection for each cell is a score that we interpreted as a pseudotime measure.

**Text S3**

**Applicability of existing metric learning algorithms**

Unfortunately, existing metric learning methods are not well suited to the challenge of synthesizing multi-modal single-cell data. Many of the considerations we discussed when comparing Schema to CCA also apply here: these methods, some of which list below, are designed to synthesize two datasets at a time, necessitating an *ad hoc* approach to integrating additional modalities. Like CCA, standard metric learning approaches do not limit the distortion of the primary modality. Setting a researcher-specified limit on this distortion is an important regularization mechanism in Schema, increasing the robustness of its results and ensuring that insights from the primary modality are not lost.

We designed Schema so it could scale to the large and ever-growing single-cell datasets. Towards that end, Schema deviates from existing metric learning approaches in computing a scaling transform and not a general affine transform. While affine transforms potentially offer more general alignment, Schema’s ability to accept arbitrary distance metrics on the secondary modalities partly compensates for this limitation on the primary modality transform. Additionally, the reach of a scaling transform is enhanced by featurizing the primary modality so that each feature represents a source of variance that is axis-aligned and orthogonal to others (e.g., using PCA or NMF). Most crucially for our needs, scaling transforms can be computed efficiently; they can be optimized using fast quadratic programming methods whereas an affine transform would need to be optimized using the much slower framework of semi-definite programming. Additionally, our choice of correlation as the measure of agreement allows for a sampling approach that further enhances scalability while producing provably accurate results (**Text S5**).

These design choices allow Schema to scale up to large single-cell datasets. We ran a set of metric learning algorithms on one of the Slide-seq samples (puck 180430_1: 22943 cells x 18133 genes), using implementations made available on the creators’ websites or in the Python package *metric_learn* [5]. We tested the following methods: neighborhood component analysis (NCA) [6]; metric learning for kernel regression (MLKR) [9]; local Fisher discriminant analysis (LFDA) [10]; large margin nearest neighbors (LMNN) [8]; and information theoretic metric learning (ITML) [7]. On a Linux server with 24 Intel Xeon 2.40 GHz cores and 386 GB RAM, each of these methods either crashed or failed to produce a meaningful output within 6 hours. In contrast, the aggregate runtime of an ensemble of Schema runs over different choices of the minimum correlation hyperparameter was 34 minutes on this dataset (**Table S1**); in each run, Schema sampled a subset of the pairwise distances between points.

**Text S4**

**Setting up the quadratic program**

We introduce some notation to condense the expressions. Define $w\in R^{k}$ where $w_{i}=u_{i}^{2}$, $\delta_{ij}\in R^{k}$with $\left( \delta_{ij} \right)_{s}=\left( \left( x_{i} \right)_{s}-\left( x_{j} \right)_{s} \right)^{2}$ (i.e. the squared elements of $x_{i}-x_{j}$) and, for convenience, let $P$ be the set of pairs of observations $P=\{\left\{ i,j \right\} :1\leq i\leq j\leq N\}$. Using the fact that the covariance between variables $X$ and $Y$ is given by $Cov\left( X,Y \right)=E\left[ XY \right]-E\left[ X \right]E[Y]$, and the variance as $Var\left( X \right)= E\left[ X^{2} \right]-E\left[ X \right]^{2}$, we can expand:

$$Cov\left( w, \rho_{l} \right)=\frac{1}{\left| P \right|}\sum_{\left\{ i,j \right\}\in P} \rho_{l}\left( x_{i}^{l}, x_{j}^{l} \right)\delta_{ij}^{T}w-\frac{1}{\left| P \right|^{2}}\left( \sum_{\left\{ i,j \right\}\in P} \delta_{ij}^{T}w \right)\left( \sum_{\left\{ i,j \right\}\in P} \rho_{l}\left( x_{i}^{l}, x_{j}^{l} \right) \right)$$

$$=\left( \frac{1}{\left| P \right|}a_{l}-\frac{1}{\left| P \right|^{2}}b_{l} \right)^{T}w$$

$$Var\left( w \right)=\frac{1}{\left| P \right|}\sum_{\left\{ i,j \right\}\in P} w^{T}\delta_{ij}\delta_{ij}^{T}w-\frac{1}{\left| P \right|^{2}}\left( \sum_{\left\{ i,j \right\}\in P} \delta_{ij}^{T}w \right)^{2}$$

$$=w^{T}\left( \frac{1}{\left| P \right|}S-\frac{1}{\left| P \right|^{2}}T \right)w$$

where $a_{l}$and $b_{l}$ are *k*-dimensional vectors that depend only on $D_{l}$; and $S$ and $T$ are $N\times k$ matrices that depend only on $D_{1}$.

Explicitly, we derive:

$$a_{l}=\sum_{\left\{ i,j \right\}\in P} \rho_{l}\left( x_{i}^{\left( l \right)},x_{j}^{\left( l \right)} \right)\delta_{ij}$$

$$b_{l}=\left( \sum_{\{i,j)\in P} \rho_{l}(x_{i}^{(l)}, x_{j}^{(l)}) \right)\sum_{\left\{ i,j \right\}\in P} \delta_{ij}$$

$$S= \sum_{\left\{ i,j \right\}\in P} \delta_{ij}\delta_{ij}^{T}$$

$$T= \left( \sum_{\left\{ i,j \right\}\in P} \delta_{ij} \right)\left( \sum_{\left\{ i,j \right\}\in P} \delta_{ij}^{T} \right)$$

We recall the general optimization problem:

|  | $\sum_{j=1}^{r} \gamma_{j}Cov(w, \rho_{j})-\alpha Var\left( w \right)- \lambda\left\vert\left\vert w-1 \right\vert\right\vert^{2}$ | (**1**) |
| --- | --- | --- |

$$subject to:$$

$$Cov\left( w, \rho_{1} \right)\geq\beta$$

$$w ⪰0$$

and the framework for quadratic programming that this needs to be mapped to:

|  | $v^{T}Qv+q^{T}v$ | (**2**) |
| --- | --- | --- |

$$subject to:$$

$$Gv⪯h$$

$$Av=b$$

where $Q$ is positive semidefinite.

The mapping from the general optimization problem (**1**) to the QP framework (**2**) is as follows:

$$v=w$$

$$Q=\frac{1}{\left| P \right|}S-\frac{1}{\left| P \right|^{2}}T+\lambda I_{k}$$

$$q= -2\lambda1- \sum_{j=1}^{r} \gamma_{l}\left( \frac{1}{\left| P \right|}a_{l}+\frac{1}{\left| P \right|^{2}}b_{l} \right)$$

We also require that $Q$ be positive semidefinite (psd). This is also straightforward to show. We can write:

$$Q=\lambda I_{k}+\frac{1}{\left| P \right|}\sum_{\left\{ i,j \right\}\in P} \left( \delta_{ij}-\mu\right)\left( \delta_{ij}-\mu\right)^{T}$$

where $\mu=\frac{1}{\left| P \right|}\sum_{\left\{ i,j \right\}\in P} \delta{}_{ij}$, so it is a sum of psd matrices.

For the linear constraint, we express $G$ as a block matrix:

$$G= \left( g^{T} 0 0 -I_{k} \right)$$

where

$$g=-\frac{1}{\left| P \right|}a_{1}-\frac{1}{\left| P \right|^{2}}b_{1}$$

Lastly, we have, for the right side of the inequality constraint, each coordinate of ***h***:

$$h_{j}=\{-\beta for j=1 0 for 2\leq j\leq k+1$$

We have no equality constraints in our optimization, so $A$ and $b$ from **(2**) are not needed.

**Text S5**

**Theoretical analysis of Schema’s scalability: concentration bounds**

Our approach is to show that, given a $\hat{u}$ that has been calculated based on a random sample, the correlation coefficient between *all* pairwise distances cannot be too different than the correlation coefficient computed on the sample. To do this, we use Chernoff bounds, which bound how far away a random variable can be from its expectation, on the covariance and variance terms of correlation coefficient given. This gives us a bound on how far away the correlation coefficient on the whole population can be from the one calculated on the sample.

Let $P$ be a random subset of all possible interactions. For now, we assume that interactions are chosen uniformly at random. Solving the optimization problem from the above section (Equation **(1))** with our sample $P$ yields $\hat{u}$, an estimator for the true optimal transform $u$. We show that $\hat{u}$ approximates $u$ well by showing that the pairwise distances of $\hat{u}(D)$ have high correlations with the secondary datasets as long as $\hat{u}$ has high correlations on the subsample.

Formally, we will guarantee, for any $\alpha, \delta>0$ and sample size at least $\left| P \right|=O\left( log \frac{\left( \frac{1}{\alpha} \right)}{\delta^{2}} \right)$,

$$\left| Corr\left( \hat{w},\rho_{j} \right)-\hat{Corr}\left( \hat{w},\rho_{j} \right) \right|<\delta with probability at least 1-\alpha,$$

where  $\hat{Corr}(\cdot, \cdot)$ is the sample correlation coefficient (i.e., the Pearson correlation computed on the sample $P$).

This is a powerful result, made possible by our restriction to scaling transforms, which are easy to analyze. First of all, note that we only need a sample-size *logarithmic* in our desired confidence level in order to get strong concentration, allowing analysis of massive RNA-seq datasets.

To begin our analysis, let $W$ be a $k\times k$psd matrix (in our specific case it will be diagonal, but this analysis will generalize to any psd matrix, which motivates the generalization to all psd matrices in future work). We also assume randomly draw pairwise differences $\delta=x_{i}-x_{j}$ , choosing these $x_{i}, x_{j}$ uniformly from the set of pairs of points in our primary dataset. Here, we focus on the correlation between the transformed dataset and the primary dataset. Analyses for correlations between the transformed data and the secondary datasets will be similar.

Consider the form of the (population) correlation:

|  | $Corr\left( W,\rho_{1} \right)=\frac{E\left[ \delta^{T}W\delta\delta^{T}\delta\right]-E\left[ \delta^{T}W\delta\right]E[\delta^{T}\delta]}{Var^{\frac{1}{2}}\left( W \right)Var^{\frac{1}{2}}(\rho_{1})}=\frac{A-BC}{DE}$ | (**3**) |
| --- | --- | --- |

If, for our samples, we can determine confidence intervals of size $2\epsilon$ for each of the terms $A, B,C,D,E$ then we can bound the distance away from the correlation on the *entire* set of pairwise distances. This distance is maximized when $A$ is as small as possible, and $B,C,D,E$ are as large as possible. So, by expanding using Taylor expansions and removing terms of size $O(\epsilon^{2})$ or smaller, we get:

$$\hat{Corr}\left( W,\rho_{1} \right)\geq\frac{\left( A-\epsilon\right)-\left( B+\epsilon\right)\left( C+\epsilon\right)}{\left( D+\epsilon\right)\left( E+\epsilon\right)}$$

$$\approx\frac{A-BC-\left( 1+B+C \right)\epsilon}{DE\left( 1+\frac{\epsilon}{D} \right)\left( 1+\frac{\epsilon}{E} \right)}$$

$$\approx\left( \frac{A-BC}{DE}-\frac{B+C+1}{DE}\epsilon\right)\left( 1-\frac{\epsilon}{D} \right)\left( 1-\frac{\epsilon}{E} \right)$$

$$\approx\left( \frac{A-BC}{DE}-\frac{B+C+1}{DE}\epsilon\right)\left( 1-\frac{\epsilon}{D}-\frac{\epsilon}{E} \right)$$

$$\approx\left( \frac{A-BC}{DE} \right)\left( 1+\frac{D+E}{DE}\epsilon\right)-\frac{B+C+1}{DE}\epsilon$$

$$=Corr\left( W,\rho_{1} \right)\left( 1-\frac{{Var}^{\frac{1}{2}}\left( W \right)+{Var}^{\frac{1}{2}}\left( \rho_{1} \right)}{{Var}^{\frac{1}{2}}\left( W \right){Var}^{\frac{1}{2}}\left( \rho_{1} \right)}\epsilon\right)-\frac{1+E\left[ \delta^{T}W\delta\right]+ E\left[ \delta^{T}\delta\right]}{{Var}^{\frac{1}{2}}\left( W \right){Var}^{\frac{1}{2}}\left( \rho_{1} \right)}\epsilon$$

Thus, for a desired overall confidence level $\eta$, the relationship between $\epsilon$ and $\eta$ is given by:

$$\epsilon=\left( \frac{{Var}^{\frac{1}{2}}\left( W \right){Var}^{\frac{1}{2}}(\rho_{1})}{\{{Var}^{\frac{1}{2}}\left( W \right)+ {Var}^{\frac{1}{2}}\left( \rho_{1} \right), 1+E\left[ \delta^{T}W\delta\right]+ E[\delta^{T}\delta]\}} \right)\eta$$

To show that we can bound each of the terms $A,B,C,D,E$$ we use *Hoeffding's inequality* to limit how far away the terms can be from their expectations. Let $X_{1},\ldots,X_{n}$ be i.i.d. random variables drawn from bounded range $[a,b]$, and set $s=b-a$, and let  $\underline{X}=\frac{1}{n}\sum_{i=1}^{n} X_{i}$. Then Hoeffding's inequality states:

$$Pr \left[ \underline{X}-EX\geq t \right]\leq exp\left( -\frac{nt^{2}}{s^{2}} \right)$$

This can be converted into giving a (one-sided) confidence interval of length $t$ by substituting the probability on the left with a desired confidence level $\alpha$, and solving for $n$, which gives a statement:

|  | $EX\geq\underline{X}-t with confidence 1-\alpha for n\geq\frac{s^{2}log\left( \frac{1}{\alpha} \right)}{t^{2}}$ | (**4**) |
| --- | --- | --- |

We begin by applying the inequality on term $A=E\left[ \delta^{T}W\delta\delta^{T}\delta\right]$ by bounding $\delta^{T}W\delta\delta^{T}\delta$. It is clear that $\left| \delta^{T}W\delta\delta^{T}\delta\right|\leq|\delta^{T}W\delta||\delta^{T}\delta|$, so we can bound each individually. Note that we can assume without loss of generality that $W$ is diagonal here, because otherwise (since it is psd), we could write $W=UDU^{T}$, where $D$ is diagonal and $U$ is unitary; setting $y=U\delta$ yields $\left| \delta^{T}W\delta\right|=\left| y^{T}Dy \right|$, and, by unitarity, $\left| \left| \delta\right| \right|=\left| \left| y \right| \right|$.

Then, by Cauchy-Schwarz:

|  | $\left\vert\delta^{T}W\delta\right\vert\leq\left\vert\sum\delta_{i}W_{ii}\delta_{i} \right\vert\leq\left\vert\left\vert W \right\vert\right\vert\left\vert\left\vert\delta\right\vert\right\vert^{2}$ | (**5**) |
| --- | --- | --- |

where $\left| \left| W \right| \right|$ is the matrix-norm, i.e. $\left| \left| W \right| \right|=\sqrt{Tr\left( W^{T}W \right)}$. So, for a diagonal matrix, $\left| \left| W \right| \right|^{2}=\sum W_{ii}^{2}$. We can bound $\left| \left| \delta\right| \right|\leq\left| \left| x_{i}-x_{j} \right| \right|\equiv diam\left( D \right) .$

Thus, $|\delta^{T}W\delta\delta^{T}\delta|\leq\left| \left| W \right| \right|{diam}^{4}\left( D \right)$.

To get a confidence interval of size $\epsilon$, we plug into (**4**), so we require:

$$N\geq\frac{\left| \left| W \right| \right|{diam}^{8}\left( D \right)log\left( \frac{1}{\alpha} \right)}{\epsilon^{2}}$$

Note that the diameter is an *extremely* coarse bound for the above bound. Morally, one can replace “diameter” with “variance”, and the user has control over $\left| \left| W \right| \right|$ by choice of hyperparameters. We also note that the sample complexity improves drastically if we focus only on *local* distances, a future area of exploration.

The same analysis can be used for terms $B$ and $C$ in (**3**), but the dependency on the diameter is not as bad for those terms, so term $A$ is the worst case.

Now, we consider the variance terms $D$ and $E$. For term $E$, note:

$$Var\left( \delta^{T}\delta\right)=E[\left( \delta^{T}\delta-E\left[ \delta^{T}\delta\right] \right)^{2}]$$

Again, $\left| \delta^{T}\delta-E\left[ \delta^{T}\delta\right] \right|$ is bounded by the maximum squared distance in the dataset $diam^{2}(D)$, so we can use the Hoeffding inequality from above in the same way.

And term $D$ takes the same form as above, but with $\delta^{T}W\delta$ instead of $\delta^{T}\delta$. As

noted in (**5**), this is a bounded random variable as well, but here with bound $\left| \left| W \right| \right|^{2}{diam}^{4}(D)$.

Thus, in order to get a uniform confidence interval across all the terms, we require:

|  | $N\geq\frac{\left\vert\left\vert W \right\vert\right\vert^{2}{diam}^{8}\left( D \right)log\left( \frac{1}{\alpha} \right)}{\epsilon^{2}}$ | (**6**) |
| --- | --- | --- |

**Text S6**

**Enriched functions and pathways for differentially expressed genes in dense granule cells**

The densely-packed granule cell genes identified by Schema are strongly enriched for signal transmission, potentially indicating greater neurotransmission activity within these cells. In particular, REACTOME ^27^ pathway enrichment analysis (top 1000 genes, mapped to human) include vesicle-mediated transport (FDR *q* = 5.11 x 10^-4^), ion-channel transport (FDR *q* = 1.82 x 10^-3^), and cellular responses to external stimuli (FDR *q* = 6.44 x 10^-15^) (**Table S2**, **Fig. S10**). An enrichment analysis of this gene set against the Gene Ontology (GO) database, performed using the GOrilla web-tool [11] and visualized using REViGO [12] also identified terms consistent with such activity: ion transport (GO:0022853, FDR *q* = 1.8 x 10^-17^), electron transfer (GO:009055, FDR *q* = 2.87 x 10^-11^) and enzyme binding (GO:0019899, FDR *q* = 2.72 x 10^-11^). (**Table S3, Fig. S9**). Interestingly, we also observed enrichment in REACTOME pathways related to autophagy (FDR *q* = 3.19 x 10^-4^), ubiquitination (FDR *q* = 1.94 x 10^-4^) and protein metabolism (FDR *q* = 3.3 x 10^-7^). In particular, we observed enrichment for the process of Neddylation (FDR *q* = 2.26 x 10^-3^), shown to have a role in nuclear protein aggregation [13,14].

**Table S1**

**Runtime comparison of Schema with CCA, SpatialDE and Trendsceek**

| **Program** | Average per sample | | |
| --- | --- | --- | --- |
|  | # of cells | # of genes | Run-time (in minutes) |
| Schema | 20823 | 17607 | 34 |
| CCA | 20823 | 17607 | 50 |
| SpatialDE | 16000 | 9000 | 244 |
| Trendsceek | 2000 | 3000 | 338 |

The values above were averaged over the three previously described mouse cerebellum samples from the Slide-seq dataset. All programs were run on a Linux server with 24 Intel Xeon 2.40 GHz cores and 386 GB RAM. Each program was allowed to use as many cores as were available; Schema, CCA and SpatialDE did so, but Trendsceek did not. The server is a shared resource and while we did periodically check it to confirm that ample system resources were available, the runtime estimates above may be influenced by the load from other programs. For Schema, the runtime includes the time for pre-processing and encompasses the complete ensemble of sub-runs on different parameter choices. The runtime for CCA also encompasses the entire pipeline: pairwise modality combinations and then a final integration. For Schema and CCA, we were able to use all the data of each sample. For SpatialDE and Trendsceek, we experimented with small subsets of data and increased the subset size until the demand on the shared resource (server) became infeasible. The subset of cells for SpatialDE and Trendsceek were randomly chosen, with an equal split between granule and non-granule cells; for both, genes were selected based on high expression variability.

**Supplementary References**

1. Pijuan-Sala B, et al. A single-cell molecular map of mouse gastrulation and early organogenesis. Nature. 2019;566:490-495.
2. Argelaguet R, et al. MOFA+: a statistical framework for comprehensive integration of multi-modal single-cell data. Genome Biol. 2020;21.
3. Hochgerner H, Zeisel A, Lönnerberg P, Linnarsson S. Conserved properties of dentate gyrus neurogenesis across postnatal development revealed by single-cell RNA sequencing. Nat Neurosci. 2018;21.
4. Bergen V, Lange M, Peidli S, Wolf FA, Theis FJ. Generalizing RNA velocity to transient cell states through dynamical modeling. Nat Biotechnol. 2020:1–7. https://doi.org/10.1038/s41587-020-0591-3.
5. de Vazelhes W, Carey C, Tang Y, Vauquier N, Bellet A. metric-learn: Metric Learning Algorithms in Python; 2019.
6. Goldberger J, Roweis S, Hinton G, Salakhutdinov R. Neighbourhood Components Analysis. In: Advances in Neural Information Processing Systems; 2004.
7. Davis JV, Kulis B, Jain P, Sra S, Dhillon IS. Information-theoretic metric learning. In: ACM International Conference Proceeding Series; 2007. <https://doi.org/10.1145/1273496.1273523>.
8. Weinberger KQ, Saul LK. Distance metric learning for large margin nearest neighbor classification. J Mach Learn Res. 2009; <https://doi.org/10.1145/1577069.1577078>.
9. Weinberger KQ, Tesauro G. Metric learning for kernel regression. 2007. In Artificial intelligence and statistics (pp. 612-619). PMLR.
10. Sugiyama M. Local fisher discriminant analysis for supervised dimensionality reduction. In: ACM International Conference Proceeding Series; 2006. <https://doi.org/10.1145/1143844.1143958>.
11. Eden E, Navon R, Steinfeld I, Lipson D, Yakhini Z. GOrilla: a tool for discovery and visualization of enriched GO terms in ranked gene lists. BMC Bioinformatics. 2009; <https://doi.org/10.1186/1471-2105-10-48>.
12. Supek F, Bošnjak M, Škunca N, Šmuc T. Revigo summarizes and visualizes long lists of gene ontology terms. PLoS One. 2011; <https://doi.org/10.1371/journal.pone.0021800>.
13. Chen Y, Neve RL, Liu H. Neddylation dysfunction in Alzheimer’s disease. J Cell Mol Med. 2012; <https://doi.org/10.1111/j.1582-4934.2012.01604.x>.
14. Maghames CM, et al. NEDDylation promotes nuclear protein aggregation and protects the ubiquitin proteasome system upon proteotoxic stress. Nat Commun. 2018; <https://doi.org/10.1038/s41467-018-06365-0>.
